# Supplementary figures and images for: Impact of developmental coordination disorder in childhood on educational outcomes in adulthood among neonatal intensive care recipients: a register-based longitudinal cohort study
Source: BMJ Open. 2023 Sep 25;13(9):e071563. doi: 10.1136/bmjopen-2023-071563 (PMC10533808; doi:10.1136/bmjopen-2023-071563)

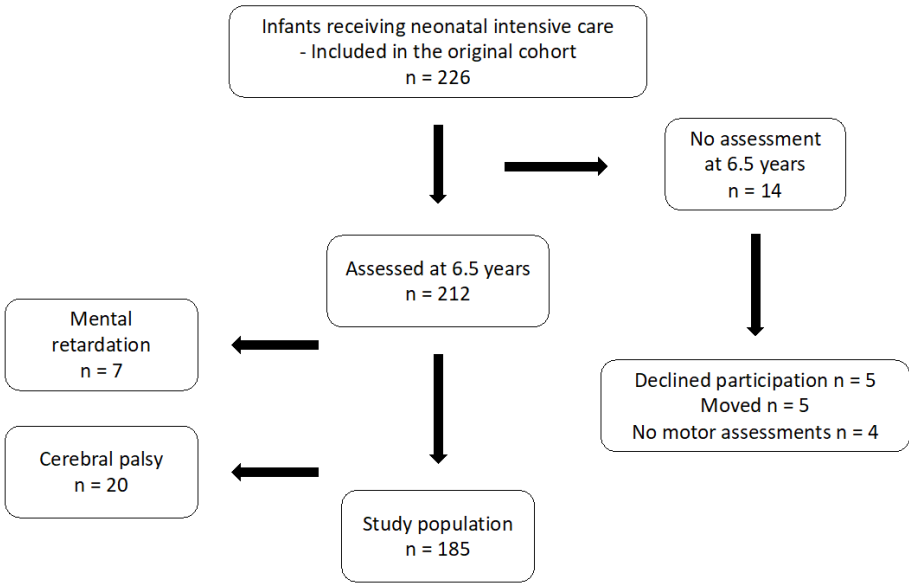

**Supplementary file 1.** Flow chart of recruitment.

Supplement: Supplementary data [file bmjopen-2023-071563supp001.pdf]
