# Supplementary material for: Impact of developmental coordination disorder in childhood on educational outcomes in adulthood among neonatal intensive care recipients: a register-based longitudinal cohort study
Source: BMJ Open. 2023 Sep 25;13(9):e071563. doi: 10.1136/bmjopen-2023-071563 (PMC10533808; doi:10.1136/bmjopen-2023-071563)
Supplement: Supplementary data [file bmjopen-2023-071563supp002.pdf]

Supplementary file 2. ICD-8/9 codes included in the variable "Diagnoses posing a risk of motor problems".

ICD-code	Diagnosis
040–046	Poliomyelitis and other enterovirus diseases of central nervous system (ICD-8)
190–199	Malignant neoplasm of other and unspecified sites (ICD-8 and ICD-9)
210–228	Benign neoplasms (ICD-8)
300–316	Neurotic disorders, personality disorders, and other nonpsychotic mental disorders (ICD-8)
320–324	Inflammatory diseases of central nervous system (ICD-8)
330–337	Hereditary and degenerative diseases of the central nervous system (ICD-9)
340–349	Other diseases of central nervous system (ICD-8)
430–438	Cerebrovascular disease (ICD-8 and ICD-9)
710–719	Arthropathies and related disorders (ICD-9)
740–759	Congenital anomalies (ICD-8 and ICD-9)
760–779	Certain conditions originating in the perinatal period (ICD-9) or certain causes of perinatal morbidity and mortality (ICD-8)
780–789	Symptoms referable to systems or organs (ICD-8)
850–854	Intracranial injury, excluding those with skull fracture (ICD-9)

Note: ICD = International Statistical Classification of Diseases and Related Health Problems - Eight/Ninth revision
