# Supplementary material for: Impact of developmental coordination disorder in childhood on educational outcomes in adulthood among neonatal intensive care recipients: a register-based longitudinal cohort study
Source: BMJ Open. 2023 Sep 25;13(9):e071563. doi: 10.1136/bmjopen-2023-071563 (PMC10533808; doi:10.1136/bmjopen-2023-071563)
Supplement: Supplementary data [file bmjopen-2023-071563supp003.pdf]

Supplementary file 3. Number of neurodevelopmental disorders registered in specialized outpatient care.

**ICD-10 Codes**		n (%)
F80-F89	Disorders of psychological development	2 (1.1)
F90-F98	Behavioral and emotional disorders with onset usually occurring in childhood and adolescence	10 (5.5)
R47-R49	Symptoms and signs involving speech and voice	2 (1.1)

F80-F89 includes: Developmental Coordination Disorder, Developmental Language Disorder, Dyslexia, Autism Spectrum Disorders and impairments in reading, writing and arithmetic.

F90-F98 includes: Attention Deficit Hyperactivity Disorder and Tic disorders.

R47-R49 includes: Dyslexia.

Note: ICD=International Statistical Classification of Diseases and Related Health Problems.
