# Supplementary material for: Impact of developmental coordination disorder in childhood on educational outcomes in adulthood among neonatal intensive care recipients: a register-based longitudinal cohort study
Source: BMJ Open. 2023 Sep 25;13(9):e071563. doi: 10.1136/bmjopen-2023-071563 (PMC10533808; doi:10.1136/bmjopen-2023-071563)
Supplement: Supplementary data [file bmjopen-2023-071563supp004.pdf]

Supplementary file 4. Educational outcomes for men and women with or without pDCD.

	**Total n=183**				**No DCD n=138**				**pDCD n=45**			
	**Men** n (%) 108 (59.0)	**Women** n (%) 75 (41.0)	**P**	***g***	**Men** n (%) 80 (58.0)	**Women** n (%) 58 (48.0)	**P**	***g***	**Men** n (%) 28 (62.2)	**Women** n (%) 17 (37.8)	**P**	***g***
**Age (yrs) at graduation from USS**												
Median	19.0	19.0	.376		19.0	19.0	.342		19.0	19.0	.882	
Min-max	19.0–26.0	19.0–26.0			19.0–22.0	19.0–26.0			19.0–26.0	19.0–24.0		
IQR	19.0-19.0	19.0-19.0			19.0-19.0	19.0-19.0			19.0-20.0	19.0-20.0		
Mean (SD)	19.3 (.9)	19.3 (1.1)		0.00	19.2 (.6)	19.2 (1.0)		0.00	19.6 (1.4)	19.8 (1.5)		0.14
Missing data, total	10 (9.3)	8 (10.7)			6 (7.5)	3 (5.2)			4 (14.3)	5 (29.4)		
No graduation	10 (9.3)	6 (8.0)	.788		6 (7.5)	3 (5.2)	.734		4 (14.3)	3 (17.7)	.692	
Absent/incomplete register data		2 (2.7)								2 (11.8)		
**USS graduation at 19 yrs**												
Yes	76 (70.4)	56 (75.7)	.431		60 (75.0)	48 (82.8)	.275		16 (57.1)	8 (50.0)	.647	
No	32 (29.6)	18 (24.3)			20 (25.0)	10 (17.2)			12 (42.9)	8 (50.0)		
Missing data		1 (1.3)								1 (5.9)		
**USS graduation at 24 yrs**												
Yes	97 (89.8)	67 (90.5)	.872		74 (92.5)	54 (93.1)	1.000		23 (82.1)	13 (81.3)	.941	
No	11 (10.2)	7 (9.5)			6 (7.5)	4 (6.9)			5 (17.9)	3 (18.8)		
Missing data		1 (1.3)								1 (5.9)		
**USS graduation at 29 yrs**												
Yes	98 (90.7)	68 (91.9)	.788		74 (92.5)	55 (94.8)	.734		24 (85.7)	13 (81.3)	.697	
No	10 (9.3)	6 (8.1)			6 (7.5)	3 (5.2)			4 (14.3)	3 (18.8)		
Missing data		1 (1.3)								1 (5.9)		
**Level of education at 28 yrs**												
Compulsory school, 9 yrs	6 (5.7)	4 (5.6)	.073		4 (5.1)	3 (5.2)	.058		2 (7.4)	1 (7.1)	.880	
Upper secondary education, ≤2 yrs	4 (3.8)	2 (2.8)			2 (2.5)	0 (0)			2 (7.4)	2 (14.3)		
Upper secondary education, 3 yrs	52 (49.1)	22 (30.6)			38 (48.1)	17 (29.3)			14 (51.9)	5 (35.7)		
Post-secondary education, <3 yrs	17 (16.0)	12 (16.7)			14 (17.7)	10 (17.2)			3 (11.1)	2 (14.3)		
Bachelor's or master's degree	27 (25.5)	32 (44.4)			21 (26.6)	28 (42.8)			6 (22.2)	4 (28.6)		
Missing data	1 (.9)	4 (5.3)			1 (1.3)				1 (3.6)	3 (17.6)		

Note: DCD=Developmental Coordination Disorder; IQR=interquartile range; Min=minimum; Max=maximum; pDCD=probable Developmental Coordination Disoder; USS=Upper Secondary School, Yrs=Years.
