# Supplementary material for: Impact of developmental coordination disorder in childhood on educational outcomes in adulthood among neonatal intensive care recipients: a register-based longitudinal cohort study
Source: BMJ Open. 2023 Sep 25;13(9):e071563. doi: 10.1136/bmjopen-2023-071563 (PMC10533808; doi:10.1136/bmjopen-2023-071563)
Supplement: Author's manuscript [file bmjopen-2023-071563.draft_revisions.pdf]

BMJ Open

BMJ Open is committed to open peer review. As part of this commitment we make the peer review history of every article we publish publicly available.

When an article is published we post the peer reviewers' comments and the authors' responses online. We also post the versions of the paper that were used during peer review. These are the versions that the peer review comments apply to.

The versions of the paper that follow are the versions that were submitted during the peer review process. They are not the versions of record or the final published versions. They should not be cited or distributed as the published version of this manuscript.

BMJ Open is an open access journal and the full, final, typeset and author-corrected version of record of the manuscript is available on our site with no access controls, subscription charges or pay-per-view fees (http://bmjopen.bmj.com).

If you have any questions on BMJ Open's open peer review process please email info.bmjopen@bmj.com

BMJ Open

The impact of developmental coordination disorder in childhood on educational outcomes in adulthood: a cohort-based longitudinal register study

Journal:	*BMJ Open*
Manuscript ID	bmjopen-2023-071563
Article Type:	Original research
Date Submitted by the Author:	02-Jan-2023
Complete List of Authors:	Persson, Isak; Uppsala University, Department of Women's and Children's Health<br>Sampaio, Filipa; Uppsala University, Department of Public Health and Caring Sciences<br>Samkharadze, Tengiz; Uppsala University, Department of Public Health and Caring Sciences<br>Ssegonja, Richard; Uppsala University, Department of Public Health and Caring Sciences<br>Johansen, Kine; Uppsala University, Department of Women's and Children's Health
Keywords:	NEONATOLOGY, MENTAL HEALTH, Developmental neurology & neurodisability < PAEDIATRICS, REGISTRIES, Neonatal intensive & critical care < INTENSIVE & CRITICAL CARE

SCHOLARONE™
Manuscripts

I, the Submitting Author has the right to grant and does grant on behalf of all authors of the Work (as defined in the below author licence), an exclusive licence and/or a non-exclusive licence for contributions from authors who are: i) UK Crown employees; ii) where BMJ has agreed a CC-BY licence shall apply, and/or iii) in accordance with the terms applicable for US Federal Government officers or employees acting as part of their official duties; on a worldwide, perpetual, irrevocable, royalty-free basis to BMJ Publishing Group Ltd ("BMJ") its licensees and where the relevant Journal is co-owned by BMJ to the co-owners of the Journal, to publish the Work in this journal and any other BMJ products and to exploit all rights, as set out in our licence.

The Submitting Author accepts and understands that any supply made under these terms is made by BMJ to the Submitting Author unless you are acting as an employee on behalf of your employer or a postgraduate student of an affiliated institution which is paying any applicable article publishing charge ("APC") for Open Access articles. Where the Submitting Author wishes to make the Work available on an Open Access basis (and intends to pay the relevant APC), the terms of reuse of such Open Access shall be governed by a Creative Commons licence – details of these licences and which Creative Commons licence will apply to this Work are set out in our licence referred to above.

Other than as permitted in any relevant BMJ Author's Self Archiving Policies, I confirm this Work has not been accepted for publication elsewhere, is not being considered for publication elsewhere and does not duplicate material already published. I confirm all authors consent to publication of this Work and authorise the granting of this licence.

For peer review only

The impact of developmental coordination disorder in childhood on educational outcomes in adulthood: a cohort-based longitudinal register study

Isak Persson[a], Filipa Sampaio[b], Tengiz Samkharadze[b], Richard Ssegonja[b,c], Kine Johansen[a]

[a] Department of Women's and Children's Health, Uppsala University, Uppsala University Children's Hospital, Uppsala, Sweden
[b] Department of Public Health and Caring Sciences, Uppsala University, Uppsala, Sweden
[c] Department of Medical Sciences, Respiratory-, Allergy- and Sleep Medicine Research Unite, Uppsala University, Uppsala University Hospital, Uppsala, Sweden

Corresponding author
Kine Johansen (ORCID 0000-0002-9212-8452), Department of Women's and Children's Health, Uppsala University, Uppsala University Hospital, 751 85 Uppsala, Sweden. Tel +46 73 904 29 94, e-mail: kine.johansen@kbh.uu.se.

Word count: 3962

Number of tables: 5

Number of figures: 1

ABSTRACT

Objectives: Developmental Coordination Disorder (DCD) is related to poorer educational outcomes among children and adolescents. Evidence on whether this association persists into adulthood is lacking. To investigate whether childhood DCD affected educational outcomes among adults, and whether this was affected by sex or a co-occurring attention deficit.

Design: Cohort-based longitudinal register study.

Setting: Neonatal intensive care (NIC) recipients born 1986-1989 at Uppsala University Children's Hospital, Uppsala, Sweden, followed through the age of 28 years.

Participants: 185 NIC recipients born 1986-1989 were included.

Primary and secondary outcome measures: At 6.5 years of age, 46 (24.6%) were diagnosed with DCD. Using register-based longitudinal data, we compared those with or without DCD in terms of: i) age at graduation from Upper Secondary School (USS), and ii) highest completed level of education at 28 years of age.

Results: Individuals with DCD were older when graduating from USS. The difference was larger among women. Although not statistically significant, having DCD and a co-occurring attention deficit affected age at graduation and level of education negatively. Women without DCD attained the highest level of education.

Conclusions: DCD among NIC recipients was associated with poorer educational outcomes in adulthood. Having DCD affected sexes differently. Early motor problems can be an indicator of persisting problems. Efforts are needed to support individuals with DCD through their academic career to mitigate negative outcomes.

Keywords
Directory; educational status; follow-up studies; longitudinal studies; motor skills disorders; intensive care, neonatal.

For peer review only

Strengths and limitations

- This paper studies the impact of developmental coordination disorder (DCD) on educational outcomes in children treated in a neonatal intensive care unit, a population with a substantially increased risk of this disorder.
- It is one of few papers that follows educational outcomes in children with DCD into adulthood, and the only one of these focusing on DCD specifically.
- Strengths of this study include the longitudinal nature of the data and the low rate of missing data inherent to national register data.
- In line with previous studies, this paper observed a statistically significant association between childhood DCD and lower levels of graduation from Upper Secondary School in Sweden, at 19 and 24 years of age, thus solidifying the belief that educational difficulties associated with DCD is not an occurrence limited to childhood and adolescence.
- We do not know if our findings are representative of the entire DCD-population, and NIC recipients may present with different problems than infants that were healthy in their neonatal period.

For peer review only

INTRODUCTION

With a prevalence of 5-6% among children [1], Developmental Coordination Disorder (DCD) affect the daily life of approximately 80 000 school-aged children in Sweden [2]. To understand how to best support those affected, there is a need to understand the socioeconomic impact of DCD over the life course. A socioeconomic measure of considerable importance is the individuals' educational level, as this closely correlates with both employment opportunities and higher wages [3].

DCD is well known to be related to poorer educational outcomes in childhood and adolescence [4–7]. However, the extent to which DCD impacts educational outcomes is complicated by the presence of other co-occurring neurodevelopmental disorders, such as attention deficit hyperactivity disorder (ADHD), dyslexia, dyscalculia, developmental language disorder and autism spectrum disorders (ASD) [1,8], which could potentially lead to poorer outcomes. Furthermore, being born premature greatly increases the risk of DCD [9], as well as of ADHD and ASD [10,11]. DCD is generally thought to be more common in boys with the male:female ratio varying between 2:1 to 7:1 in the literature [1]. A potential sex influence on symptom severity in children with DCD exists [12,13], where girls with coordination problems report poorer self-perception, and poorer academic competence [13].

Compared to typically developing peers, children with DCD have an increased risk of language skills difficulties [14], impaired numerical cognition abilities [15], and executive dysfunction [1,15]. DCD is also well known to affect handwriting [1], which might further affect the individual's ability to succeed at school. Considering academic outcomes, Harowell et al showed that 16-year-old English adolescents with DCD achieved significantly lower grades on national exams than those without the disorder [5]. In accordance, both Rasmussen & Gillberg and Landgren et al found that individuals with ADHD and a co-occurring DCD exhibited poorer educational outcomes than individuals diagnosed with ADHD alone [6,7,16]. Looking beyond DCD, there are several other factors that may affect educational outcomes. In childhood and adolescence, prematurity and ADHD are well-known to be associated with poorer educational outcomes [17,18]. Additionally, socioeconomic background, parental educational level, migration background and income affect educational outcomes [19].

In Sweden, education is tax-financed and offered free-of-charge. After 9 years of compulsory schooling, i.e. at approximately 15 years of age, the majority of the adolescents choose to start Upper Secondary School (USS), an elective preparatory or vocational program lasting for three years [20]. Graduation from USS is a prerequisite for all higher education and is commonly requested in the labor market for employment, even for occupations where

For peer review only - http://bmjopen.bmj.com/site/about/guidelines.xhtml

educational requirements have traditionally been low [21]. In 2021, 31.4% of the Swedish labor force without graduation from USS were unemployed, compared to 7.8% among those who had graduated from USS without completing any post-secondary education [22]. According to the Delegation for the Employment of Young People and Newly Arrived Migrants (DUA), 65% of all students born in 1989 graduated from USS before age 20 and 91% before age 25 [23]. There was also a considerable gender difference; at 25 years of age, 12% of men lacked graduation compared to 6% of women [23]. The societal costs of one individual not finishing USS in Sweden is estimated at 163,000 € [24]. In 2021, 30% of the population aged 25-64 had attained at least a bachelor's degree [25], and the average age to reach this level was 28 years [3]. At age 40, 39% of women and 26% of men had attained at least a bachelor's degree [26].

Although poorer educational outcomes of individuals with DCD is well-documented in childhood and adolescence, there is a paucity of studies examining if this association persists into adulthood. Therefore, using a register-based longitudinal design, we explored how DCD diagnosed in childhood among a cohort of Swedish neonatal intensive care (NIC) recipients, affected educational outcomes in adulthood. Additionally, we investigated whether sex or a co-occurring attention deficit affected educational outcomes.

MATERIAL AND METHODS

Study design and participants

In this cohort-based longitudinal register study, we used data from a cohort of neonatal care recipients born at University Children's Hospital in Uppsala, Sweden, between the 1st of January 1986 and the 30th of April 1989. All surviving infants (n=246) treated at the neonatal intensive care unit during this period, who were residents in Uppsala County, were enrolled in a longitudinal follow-up study [27]. For a detailed description of the study population please see Montgomery et al [28]. The children underwent repeated assessments from estimated date of birth up to 10 years of age [29]. At 6.5 years, 212 children were assessed using the Standardized Test of Motor Impairment (TOMI) [30] and the Motor-Perceptual Development (MPU) [29,31]. The assessment at 6.5 years was used as the baseline for the present study. The children have previously been retrospectively diagnosed as having: no motor deviations (n = 68), motor delay or motor problem (n = 71) or DCD (n = 46) [29]. For the purpose of the present study, those diagnosed with cerebral palsy (n = 20) and mental retardation (n = 7) were excluded. This left us with 185 participants eligible for participation (Figure 1). During

For peer review only

the initial study period, Uppsala County had 280 000 inhabitants with a slightly younger and slightly more highly educated population than the national average [28].

This study adhered to the STROBE cohort checklist recommendations [32].

<<< Figure 1. Insert here >>>

Data sources

We collected data on sex, gestational age and outcome of the motor assessment at 6.5 years of age from the original cohort study. In 2019, data on educational outcomes was retrieved from the 'Longitudinal Integrated Database for Health Insurance and Labor Market Studies' (LISA) [33], as well as data on diagnoses and healthcare consumption from the 'National Patient Register' [34]. Using the participants' personal number, data was extracted from the abovementioned registers. The National Board of Health and Welfare matched and anonymized the data before delivering it to the project leader (last author). Data were available for the years 2002-2018.

Outcome variables

The LISA-register integrates yearly information about education, employment and demographic characteristics. Education level is reported on an ordinal scale ranging from less than 9 years compulsory school to a licentiate's or doctor's degree [35]. This variable was used to create: i) a continuous variable indicating at what age the participants graduated USS, using the year of graduation from USS reported in the register; ii) three categorical variables indicating whether the participants had graduated USS at 19, at 24 and at 29 years of age (yes/no); and iii) one categorical variable indicating the educational level at 28 years of age. The age-specific binary variables at 19 and 24 years of age were constructed as data on the average graduation rates in the Swedish population born in 1989 was available for those ages [23], while 29 years was the last point of measurement that allowed analysis of the whole sample. As the mean age to attain a bachelor's degree in Sweden is 28 years [3] this age was chosen when investigating educational level.

Exposure variables

The exposure variables were categorical variables indicating: i) whether the participants were diagnosed as having DCD or not at the motor assessment at 6.5 years of age (yes/no) [29], ii) their sex (man/woman), and iii) if the participant had a co-occurring attention deficit (yes/no).

For peer review only

Covariates

Sex, gestational age, number of diagnoses posing a risk of motor problems, and total number of days in hospital during the first year of life, were added as covariates in the logistic regression model, as these could affect both the risk of DCD and the educational outcome. The diagnoses were coded according to International Statistical Classification of Diseases and Related Health Problems - eighth/ninth revision (ICD-8/9) into groups of diagnoses. Hence, we could not identify specific diagnoses or disorders. Instead, we categorized these groups of diagnoses into whether they posed an increased risk of motor problems and created a continuous variable indicating the total number of diagnoses. For a detailed list of ICD-codes included in the variable, please see supplementary file 1. Additionally, we counted the total number of days in inpatient care during the first year of life.

Missing data

For two female participants assessed with DCD at 6.5 years, all educational data was missing in the LISA-register. These were excluded from all statistical analyses, leaving a total sample of 183 participants. Additionally, two other women with DCD had incomplete register data. Data from these participants was used when values could be assigned to the categorical outcome variables indicating graduation from USS but were otherwise excluded. When analyzing level of education at 28 years of age, four participants had emigrated and were lost to follow up.

When performing the regression analysis, data was missing regarding diagnoses and healthcare consumption during the first year of life for nine participants. To minimize loss of data, we imputed the data using multiple imputation by chained equations. Five datasets were imputed using all available variables (i.e., all extracted data retrieved from the sources in 2.2.) as predictors.

Statistical analysis

Descriptive statistics are presented for the covariates and outcome variables comparing those diagnosed with DCD at 6.5 years of age and those without. Additionally, we performed two sub-group analyses exploring if educational outcomes among the participants diagnosed with DCD at 6.5 years of age differed by: i) sex and ii) having a co-occurring attention deficit. Differences in group characteristics were analyzed with chi-square tests for categorical variables. Mann Whitney U test was used to analyze differences between groups for continuous variables. Logistic regression was used to investigate the relationship between DCD and educational outcomes. A two-tailed p-value of <0.05 was considered statistically

For peer review only - http://bmjopen.bmj.com/site/about/guidelines.xhtml

significant. All analyses were performed using IBM SPSS Statistics 26 for Windows (SPSS Inc., Chicago, IL, USA).

Patient and public involvement

No patient or public involvement.

Ethical approval

Approval was given by the Swedish Ethical Review Authority (reg. no. 2019-04119). The register data were delivered anonymized but linked to the participants via unique identification numbers administered by the Swedish Board of Health and Welfare. All data were stored at the Department of Women's and Children's Health at Uppsala University and on password protected servers held by the University. Data were handled in accordance to the General Data Protection Regulation and Swedish legislation. The processing of personal data was registered in Uppsala University's register of personal data processing and monitored by the Data Protection Officer.

RESULTS

Sample characteristics

The characteristics of the study participants are presented in Table 1. The majority of the 183 participants were born moderate to late preterm (gestational week (GW) 32-36; n = 70, 38.3%) or full-term (GW >37; n = 62, 33.9%). The corresponding numbers for the very preterm (GW 28-31) and extremely preterm (GW 22-27) were 42 (23.0%) and 9 (4.9%), respectively. Slightly more than half of the participants were men (n = 108, 58.4%). On average, the participants had four diagnoses posing a risk of motor problems (min-max 0-10) and spent 24 days in hospital during the first year of life (min-max 0-200).

When comparing the sample characteristics, no statistically significant differences were found between those diagnosed with DCD and those without DCD at 6.5 years of age. The DCD-group contained slightly more men (n = 28, 62.2% vs n = 80, 58.0%) and spent on average 2.5 more days in hospital during their first year of life (26 vs 23.5 days). When comparing the distribution between gestational age groups, it can be noted that the DCD group had a higher relative count of extremely preterm births (n = 4, 8.9%) compared to those without DCD (n = 5, 3.6%), although not statistically significant (p = .502).

Few participants had any diagnosis of a neurodevelopmental disorder recorded in specialized outpatient care (supplementary file 2).

For peer review only

Table 1. Sample characteristics.

	Total	No DCD	DCD	P
	n (%)	n (%)	n (%)	
	183 (100)	138 (75.4)	45 (24.6)	
**Sex**				.615
Men	108 (59.0)	80 (58.0)	28 (62.2)	
Women	75 (41.0)	58 (42.0)	17 (37.8)	
**Gestational age**				.502
22–27 GW	9 (4.9)	5 (3.6)	4 (8.9)	
28–31 GW	42 (23.0)	31 (22.5)	11 (24.4)	
32–36 GW	70 (38.3)	55 (39.9)	15 (33.3)	
Full term	62 (33.9)	47 (34.1)	15 (33.3)	
**Number of diagnoses posing a risk of motor problems**				.891
Median	4.00	4.00	4.00	
Min-max	0–10	0–10	1–9	
Mean (SD)	4.0 (1.9)	4.0 (1.9)	4.0 (1.9)	
**Number of days in hospital during first year of life**				.489
Median	24.00	23.50	26.00	
Min-max	0–200	1–200	0–168	
Mean (SD)	32.1 (30.5)	30.8 (29.2)	35.9 (34.1)	

Note: DCD=Diagnosed with Developmental Coordination Disorder; GW=gestational weeks, Min=minimum; Max=maximum; SD=Standard Deviation.

Educational outcomes

Participants diagnosed with DCD at 6.5 years of age exhibited the lowest educational level on all variables (Table 2). There were statistically significant differences between groups for age at graduation from USS (p = .002) and the proportion of participants that had graduated at 19 (p = .002) and 24 (p = .044) years of age. In the whole sample, 16 participants had not finished USS (8.7%). Among these, seven were diagnosed with DCD (15.6%) compared to nine without DCD (6.5%). The mean age at graduation from USS for the whole sample was 19.3 years of age (±1.0). There was a statistically significant difference between those diagnosed with and without DCD (p = .022), where those without DCD graduated at 19.2 years compared to 19.6 years among those with DCD. At 19 years of age, 78.3% of those without DCD had graduated from USS, compared to 54.5% among those with DCD (p = .002). At 24 years of age, the corresponding numbers were 92.8% and 84.1% (p = .044), respectively. In total, 33% of the participants had attained a bachelor's or a master's degree, and there was no statistically significant difference between the groups (p = .080). However, 35.8% of those without DCD had attained a bachelor's or a master's degree at this age, while the corresponding number for those with DCD was 24.4%.

For peer review only

Table 2. Educational outcomes for participants with and without DCD.

	Total	No DCD	DCD	P
	n (%)	n (%)	n (%)	
	183 (100)	138 (75.4)	45 (24.6)	
**Age (yrs) at graduation from USS**				.022
Median	19.00	19.00	19.00	
Min-max	19–26	19–26	19–26	
Mean (SD)	19.3 (1.0)	19.2 (0.8)	19.6 (1.4)	
Missing data, total	18 (9.8)	9 (6.5)	9 (20.0)	
No graduation	16 (8.7)	9 (6.5)	7 (15.6)	
Absent/incomplete register data	2 (1.1)	0 (0)	2 (4.4)	
**USS graduation at 19 yrs**				.002
Yes	132 (72.5)	108 (78.3)	24 (54.5)	
No	50 (27.5)	30 (21.7)	20 (45.5)	
Missing data	1 (0.5)	0 (0)	1 (2.2)	
**USS graduation at 24 yrs**				.044
Yes	164 (90.1)	128 (92.8)	36 (81.8)	
No	18 (9.9)	10 (7.2)	8 (18.2)	
Missing data	1 (0.5)	0 (0)	1 (2.2)	
**USS graduation at 29 yrs**				.069
Yes	166 (91.2)	129 (93.5)	37 (84.1)	
No	16 (8.8)	9 (6.5)	7 (15.9)	
Missing data	1 (0.5)	0 (0)	1 (2.2)	
**Level of education at 28 yrs**				.080
Compulsory school, 9 yrs	10 (5.6)	7 (5.1)	3 (7.3)	
Upper secondary education, ≤2 yrs	6 (3.4)	2 (1.5)	4 (9.8)	
Upper secondary education, 3 yrs	74 (41.6)	55 (40.1)	19 (46.3)	
Post-secondary education, <3 yrs	29 (16.3)	24 (17.5)	5 (12.2)	
Bachelor's or master's degree	59 (33.1)	49 (35.8)	10 (24.4)	
Missing data	5 (2.7)	1 (0.7)	4 (8.9)	

Note: DCD=Developmental Coordination Disorder; Yrs=Years; Min=minimum; Max=maximum; SD=Standard Deviation; USS=Upper Secondary School.

Although the proportion of the changes in the dependent variable, i.e., graduation from USS, explained by the covariates included in the regression models were quite small (Table 3), the negative contribution of a DCD-diagnosis was significant for graduation at both 19 and 24 years of age, in both unadjusted and adjusted models. Those diagnosed with DCD at 6.5 years of age had 33.3% of the odds of graduating USS at age 19 compared to those without DCD ($p = .003$; 95% CI: 0.16-0.68). The odds of graduating from USS remained relatively stable (31.5-34.5%) when adjusting for sex, gestational age, number of diagnoses posing a risk of motor problems and the number of hospital days during the first year of life.

For peer review only

Table 3. Logistic regression results for prediction of graduation at 19, 24 and 29 years of age.

Covariates	R^2	B	OR	95% CI	P
**USS graduation at 19 yrs**					
**Unadjusted**	.069				
DCD (DCD vs no DCD*)		-1.099	.333	(0.16, 0.68)	.003
**Adjusted**	.112-.136**				
DCD (DCD vs no DCD*)		-1.154	.315	(0.15, 0.66)	.002
Sex (men vs women*)		-0.208	.812	(0.40, 1.66)	.568
Gestational age					
22–27 GW vs full term*		1.370	3.937	(0.50, 30.98)	.193
28–31 GW vs full term*		0.726	2.066	(0.60, 7.08)	.248
32–36 GW vs full term*		0.600	1.823	(0.72, 4.57)	.201
Number of					
Diagnoses posing a risk of motor problems		-0.204	.815	(0.65, 1.03)	.082
Days in hospital during first year of life		-0.007	.993	(0.98, 1.01)	.426
**USS graduation at 24 yrs**					
**Unadjusted**	.045				
DCD (DCD vs no DCD*)		-1.045	.352	(0.13, 0.96)	.041
**Adjusted**	.089-.102**				
DCD (DCD vs no DCD*)		-1.134	.322	(0.12, 0.90)	.031
Sex (men vs women*)		.100	1.105	(0.39, 3.12)	.850
Gestational age					
22–27 GW vs full term*		19.450	280 x 10^6	(0.00, 0.00)	.999
28–31 GW vs full term*		-.349	0.705	(0.13, 4.39)	.763
32–36 GW vs full term*		-.142	1.017	(0.26, 4.00)	.980
Number of					
Diagnoses posing a risk of motor problems		-.142	.868	(0.63, 1.19)	.377
Days in hospital during first year of life		.001	1.001	(0.98, 1.03)	.936
**USS graduation at 29 yrs**					
**Unadjusted**	.040				
DCD (DCD vs no DCD*)		-.998	.369	(0.13, 1.06)	.063
**Adjusted**	.076-.087**				
DCD (DCD vs no DCD*)		-1.066	.345	(0.18, 1.01)	.053
Sex (men vs women*)		.012	1.012	(0.34, 3.02)	.983
Gestational age					
22–27 GW vs full term*		18.959	171 x 10^6	(0.00, 0.00)	.999
28–31 GW vs full term*		-.349	.705	(0.10, 4.81)	.721
32–36 GW vs full term*		.111	1.118	(0.27, 4.70)	.879
Number of					
Diagnoses posing a risk of motor problems		-.149	.862	(0.62, 1.20)	.372
Days in hospital during first year of life		.008	1.008	(0.98, 1.04)	.600

For peer review only

Note: R^2 = Neglekerke's R^2; OR = Odds Ratio CI=Confidence Interval; yrs=Years; DCD=Developmental Coordination Disorder; GW=gestational weeks.
*Reference ** Range denotes the variance of the value of R^2 between different imputations

Educational outcomes by sex

At 19 years of age, the proportion of women with DCD (n = 8, 50.0%) who graduated from USS were statistically significantly lower compared to women without DCD (n = 48, 82.8%; *p* = .017) (Table 4). The similar tendency was seen between men with or without DCD, although not statistically significant. No other statistically significant differences were found in educational outcomes between men and women with or without DCD, respectively. However, when investigating differences in level of education at 28 years of age more women without DCD had attained a bachelor's or a master's degree (n = 28, 48.3%) compared to women with DCD (n = 4, 28.6%), men without DCD (n = 21, 26.6%), and men with DCD (n = 6, 22.2%).

Table 4. Educational outcomes for men and women with or without DCD.

	Men n=108			Women n=75		
	**No DCD** n (%) 80 (74.1)	**DCD** n (%) 28 (25.9)	**P**	**No DCD** n (%) 58 (77.3)	**DCD** n (%) 17 (22.7)	**P**
**Age (yrs) at graduation from USS**			.157			.067
Median	19.00	19.00		19.00	19.00	
Min-max	19–22	19–26		19–26	19–24	
Mean (SD)	19.2 (.6)	19.6 (1.4)		19.2 (1.0)	19.8 (1.5)	
Missing data, total	6 (7.5)	4 (14.3)		3 (5.2)	5 (29.4)	
No graduation	6 (7.5)	4 (14.3)		3 (5.2)	3 (17.6)	
Absent/incomplete register data	0 (0)	0 (0)		0 (0)	2 (11.8)	
**USS graduation at 19 yrs**			.075			.017
Yes	60 (75.0)	16 (57.1)		48 (82.8)	8 (50.0)	
No	20 (25.0)	12 (42.9)		10 (17.2)	8 (50.0)	
Missing data	0 (0)	0 (0)		0 (0)	1 (5.9)	
**USS graduation at 24 yrs**			.148			.168
Yes	74 (92.5)	23 (82.1)		54 (93.1)	13 (81.3)	
No	6 (7.5)	5 (17.9)		4 (6.9)	3 (18.8)	
Missing data	0 (0)	0 (0)		0 (0)	1 (5.9)	
**USS graduation at 29 yrs**			.281			.111
Yes	74 (92.5)	24 (85.7)		55 (94.8)	13 (81.3)	
No	6 (7.5)	4 (14.3)		3 (5.2)	3 (18.8)	
Missing data	0 (0)	0 (0)		0 (0)	1 (5.9)	
**Level of education at 28 yrs**			.665			.082
Compulsory school, 9 yrs	4 (5.1)	2 (7.4)		3 (5.2)	1 (7.1)	
Upper secondary education, ≤2 yrs	2 (2.5)	2 (7.4)		0 (0)	2 (14.3)	

For peer review only

Upper secondary education, 3 yrs	38 (48.1)	14 (51.9)	17 (29.3)	5 (35.7)
Post-secondary education, <3 yrs	14 (17.7)	3 (11.1)	10 (17.2)	2 (14.3)
Bachelor's or master's degree	21 (26.6)	6 (22.2)	28 (48.3)	4 (28.6)
Missing data	1 (1.3)	1 (3.6)	0 (0)	3 (17.6)

Note: DCD=Developmental Coordination Disorder; Yrs=Years; Min=minimum; Max=maximum; SD=Standard Deviation; USS=Upper Secondary School.

Educational outcomes with or without a co-occurring attention deficit

Approximately half of the participants with DCD were diagnosed with a co-occurring attention deficit at 6.5 years of age (n = 22, 48.9%) (Table 5). There were no statistically significant differences in educational outcomes between those diagnosed with DCD and an attention deficit and those with DCD without an attention deficit. However, at all ages, fewer of those with DCD and an attention deficit graduated from USS compared to those with DCD alone. The differences were most evident at 19 (42.9% vs 65.2%; p = .137) and 24 years of age (72.7% vs 90.9%; p = .240). At 29 years of age, 22.7% (n = 17) of the participants diagnosed with DCD and an attention deficit at 6.5 years of age had not graduated from USS. At 28 years of age, five participants in both groups had a degree from higher education (23.8% vs 25.0%).

Table 5. Educational outcomes for participants with DCD with or without a co-occurring attention deficit.

	DCD	DCD -	DCD +	P
	n (%)<br>45 (100)	n (%)<br>23 (51.1)	n (%)<br>22 (48.9)	
**Age (yrs) at graduation from USS**				.227
Median	19.00	19.00	19.00	
Min-max	19–26	19–24	19–26	
Mean (SD)	19.6 (1.4)	19.5 (1.2)	19.9 (1.8)	
Missing data, total	9 (20.0)	3 (13.0)	6 (27.3)	
No graduation	7 (15.6)	2 (8.7)	5 (22.7)	
Absent/incomplete data	2 (4.4)	1 (4.3)	1 (4.6)	
**USS graduation at 19 yrs**				.137
Yes	24 (54.5)	15 (65.2)	9 (42.9)	
No	20 (45.5)	8 (34.8)	12 (57.1)	
Missing data	1 (2.2)	0 (0)	1 (4.5)	
**USS graduation at 24 yrs**				.240
Yes	36 (81.8)	20 (90.9)	16 (72.7)	
No	8 (18.2)	2 (9.1)	6 (27.3)	
Missing data	1 (2.2)	1 (4.3)	0 (0)	
**USS graduation at 29 yrs**				.412
Yes	37 (84.1)	20 (90.9)	17 (77.3)	

For peer review only

No	7 (15.9)	2 (9.1)	5 (22.7)	
Missing data	1 (2.2)	1 (4.3)	0 (0)	
**Level of education at 28 yrs**				.755
Compulsory school, 9 yrs	3 (7.3)	1 (5.0)	2 (9.5)	
Upper secondary education, ≤2 yrs	4 (9.8)	1 (5.0)	3 (14.3)	
Upper secondary education, 3 yrs	19 (46.3)	11 (55.0)	8 (38.1)	
Post-secondary education, <3 yrs	5 (12.2)	2 (10.0)	3 (14.3)	
Bachelor’s or master’s degree	10 (24.4)	5 (25.0)	5 (23.8)	
Missing data	4 (8.9)	3 (13.0)	1 (4.5)	

Note: DCD=Developmental Coordination Disorder; DCD – = DCD without an attention deficit; DCD + = DCD and an attention deficit; Yrs=Years; Min=minimum; Max=maximum; SD=Standard Deviation; USS=Upper Secondary School.

For peer review only

DISCUSSION

Using longitudinal individual level data from national registers on a cohort of NIC recipients, we found that NIC recipients had an educational level similar to that of the national average at 19 and 24 years of age [23]. However, fewer individuals diagnosed with DCD at 6.5 years of age graduated USS, and those who did, generally did so at a later age. When the results were not statistically significant, the same direction of results were observed. Additionally, a similar, but non-significant direction of results was seen, where women with DCD as well as participants with DCD and a co-occurring attention deficit consistently exhibited the poorest educational outcomes.

DCD and educational outcomes

Adding to previous research, where DCD has shown to negatively affect educational outcomes during childhood and adolescence [4–7], our results indicate that this effect persists into adulthood. In our sample of NIC recipients, childhood DCD was associated with lower odds of, and a higher age at, graduation from USS. Further, being diagnosed with DCD predicted poorer educational outcomes independent of other covariates known to affect education, such as prematurity and sex [23,26,36]. A difference was also seen in completion of post-secondary education, although not statistically significant, where 33.1% of the NIC recipients without DCD compared to 24.4% of those with DCD had attained a bachelor’s or a master’s degree at 28 years of age. In Sweden, 30% of the general population (aged 25-64) attains a bachelor’s degree [25], on average at age 28 [3]. Poor educational outcomes are linked to higher unemployment rates and lower wages [3,21,22], and are costly to both the individual and society [24]. Our findings indicate that supporting these children through their academic career could help mitigate the impact of DCD on individuals and their families and reduce the economic burden of DCD on society.

Interaction between DCD and sex

Swedish women have considerably higher rates of graduation from USS and completion of post-secondary education than men [23,26]. When we investigated the educational outcomes within the sexes, we found that less women with DCD had graduated from USS at 19 years of age compared to women without DCD. In contrast, female NIC recipients without DCD performed similar to, or better than, the general population [23]. A similar pattern was found in the level of education at 28 years of age. Female NIC recipients without DCD had to a larger extent attained a bachelor's or a master's degree compared to all other groups. Although it is difficult to draw any conclusion from this small high-risk sample, our findings are in line with previous research indicating that women with DCD are at an increased risk of experiencing adverse outcomes of their motor problems [12,13]. Our results highlight the need for future research to investigate if and how motor problems impact men and women differently.

Interaction between DCD and attention deficits

Co-occurring neurodevelopmental disorders such as ADHD are known to entail poorer outcomes in a variety of domains [6,7,16]. Although no statistically significant differences were observed in our sample when comparing educational outcomes between participants with DCD with or without a co-occurring attention deficit, the former group consistently exhibited poorer outcomes. A similar trend was found by Landgren et al, 36% of Swedes diagnosed with ADHD and DCD at 6.6 years of age still lacked graduation from USS in their early thirties [7]. In our study, 22.7% of the participants diagnosed with DCD and an attention deficit at 6.5 years of age had not graduated from USS at age 29. This could be compared to 9.1% of those with DCD alone. As previous studies have shown, the combination of ADHD and DCD is particularly ominous, as this group seems to experience substantially worse outcomes than individuals with either ADHD or DCD alone [7,8,16]. Even though DCD is as common as more well-known neurodevelopmental disorders such as ADHD or ASD [8], the awareness and knowledge of DCD are limited among healthcare and educational professionals [37,38]. This lack of awareness often results in extensive delay to diagnosis and access to intervention. To promote the health and well-being of children with DCD, initiatives to raise awareness about the disorder are needed [39].

DCD and prematurity

When considering the generalizability of our results, we need to consider the special properties of the cohort. All participants are NIC recipients and two-thirds were born

For peer review only - http://bmjopen.bmj.com/site/about/guidelines.xhtml

premature (GW < 37). Prematurity is associated with poorer cognitive and academic outcomes [36,40–42], and an increased risk of not reaching curriculum goals during primary school [17,42,43]. Prematurity also is associated with an increased risk of DCD [9]. Therefore, it is not surprising that DCD was diagnosed in 24.6% (n = 45) of the participants, nearly five times more often than in the general population [1]. However, considering that approximately 10% of the global population is born premature [44] and prematurity increases the risk of DCD by three- to fourfold [9], children born premature are a substantial part of the DCD-population. Given that being born prematurely increases the risk of adverse outcomes in motor, cognitive, and behavioral domains, multidisciplinary teams are needed to comprehensively assess children's strengths and difficulties to be able to provide tailored interventions to optimize development, divert negative pathways, as well as mitigate negative outcomes [45]. Our results highlight the need of assessing motor performance in children born preterm, as it is an important predictor of long-term negative outcomes [6,7].

Strengths and limitations

Strengths of this study include the longitudinal nature of the data and the low rate of missing data inherent to national register data. Additionally, in contrast to many other studies on DCD, the definition according to the Diagnostic and Statistical Manual of Mental Disorders, 4th edition was utilized, which increased the reliability of the diagnosis. However, the small sample size may have impacted on the study´s ability to detect true differences, hence, the results need to be interpreted with caution. This is especially true for the sub-group analyses, where the groups became very small. Furthermore, access to a population-matched control group would have increased the validity of the results. When it comes to educational outcomes, there are also possible covariates that we did not have access to, and hence, could not include in the regression analysis. Most notably, socioeconomic background, parental education [19], birth weight [46], and co-occurring neurodevelopmental disorders. Knowing that co-occurring neurodevelopmental disorders affect educational outcomes, we wanted to control for these in the analysis. However, according to the National Patient Registry data, very few individuals were diagnosed with such disorders (supplementary file 2). This is interesting in itself, given the increased risk of neurodevelopmental disorders among preterm infants [9–11], but it also made it difficult for us to investigate the effect of other neurodevelopmental disorders on educational outcomes.

The generalizability of our results might be limited by the properties of the study population and the age at follow-up. We do not know if our findings are representative of the

For peer review only

entire DCD-population, and NIC recipients may present with different problems than infants that were healthy in their neonatal period. Further, given the huge advances in neonatal care since the late 80-ties, we do not know if this affects outcomes. However, there is evidence that the increased survival rates among the extremely premature has not been accompanied by decreased rates of neurodevelopmental disorders and educational difficulties [47,48]. Thus, there is reason to believe our results would still be relevant today. The age at follow-up could also affect our results, mainly the results regarding the highest level of education. Given that we retrieved the data when the participants were 29-32 years of age, it is likely that study participants would continue to graduate later in life.

Conclusions

This study points towards DCD and DCD with a co-occurring attention deficit in childhood as being associated with poorer educational outcomes in NIC recipients in adulthood. While further research is needed to establish if this holds true for the entire DCD-population, our study points to motor problems during childhood as an indicator of later adverse outcomes. To ameliorate these outcomes, efforts are needed to increase knowledge and awareness of DCD among healthcare and educational professionals as well as providing intervention in the school setting to support children with DCD through their academic career. Finally, our study inconclusively points towards a DCD-diagnosis having a larger negative effect on educational outcomes in women, which needs to be explored in future research.

Acknowledgements

We want to acknowledge the children and parents who participated in the initial follow-up study. Additionally, we want to thank Elvira Hemgren (PT), Kristina Persson (PT, PhD) and Bo Strömberg (MD, PhD) for their important work in collecting the baseline data, as well as Jill Zwicker (OT, PhD) and Amanda Kirby (MD, PhD) for their valuable advice during the initial phases of the project.

Author contributions

KJ acquired funding, conceptualized and designed the study with support from FS and RS. IP analyzed and managed the data and drafted the initial manuscript. FS, TS, RS and KJ supported data analysis. KJ revised the manuscript. All authors critically reviewed the manuscript, contributed to drafts and approved the final version. We attest all authors meet ICMJE requirements for authorship. KJ acts as guarantor.

For peer review only

Funding

This work was supported by the Gillbergska Foundation, Uppsala University Hospital through the Medical Training and Research Agreement Funds (ALF); and Uppsala University through the Hedström Foundation and the Larsson Foundation.

Competing interests

None declared.

Patient consent for publication

Not applicable.

Data availability statement

Data cannot be shared publicly because it includes potentially identifying and sensitive patient information, therefore access is limited to by request by the Swedish Ethical Review Authority (reg. no. 2019-04119). Data are available from Uppsala University, Legal Affairs Division (contact via e-mail: registrator@uu.se), for researchers who meet the criteria for access to confidential data.

Ethics approval statement

Approval was given by the Swedish Ethical Review Authority (reg. no. 2019-04119). The register data were delivered anonymized but linked to the participants via unique identification numbers administered by the Swedish Board of Health and Welfare. All data were stored at the Department of Women's and Children's Health at Uppsala University and on password protected servers held by the University. Data were handled in accordance to the General Data Protection Regulation and Swedish legislation. The processing of personal data was registered in Uppsala University's register of personal data processing and monitored by the Data Protection Officer.

For peer review only

References

1. Blank R, Barnett AL, John Cairney, et al. International clinical practice recommendations on the definition, diagnosis, assessment, intervention, and psychosocial aspects of developmental coordination disorder. Dev Med Child Neurol 2019;61:242-85. https://doi.org/10.1111/dmcn.14132
2. Statistics Sweden. Population by region, marital status, age and sex. Year 1968 - 2020. Retrieved from: https://www.statistikdatabasen.scb.se/pxweb/sv/ssd/START__BE__BE0101__BE0101A/BefolkningNy/table/tableViewLayout1/. Accessed June 13, 2022.
3. OECD. Education at a Glance 2021: OECD Indicators. OECD Publishing, Paris. https://doi.org/10.1787/b35a14e5-en
4. Cantell MH, Smyth MM, Ahonen TP. Two distinct pathways for developmental coordination disorder: Persistence and resolution. Hum Mov Sci 2003;22:413–31. https://doi.org/10.1016/j.humov.2003.09.002.
5. Harrowell I, Hollén L, Lingam R, et al. The impact of developmental coordination disorder on educational achievement in secondary school. Res Dev Disabil 2018;72:13–22. https://doi.org/10.1016/j.ridd.2017.10.014
6. Landgren V, Fernell E, Gillberg C, et al. Deficits in attention, motor control and perception childhood to age 30 years: prospective case-control study of outcome predictors. BMJ Open 2022;12:e54424. https://doi.org/10.1136/bmjopen-2021-054424
7. Landgren V, Fernell E, Gillberg C, et al. Attention-deficit/hyperactivity disorder with developmental coordination disorder: 24-year follow-up of a population-based sample. BMC Psychiatry 2021;21:161. https://doi.org/10.1186/s12888-021-03154-w.
8. Cleaton MA, Kirby A. Why do we find it so hard to calculate the burden of neurodevelopmental disorders. J Child Dev Disord 2018;4:10. https://doi.org/10.4172/2472-1786.100073
9. Spittle AJ, Orton J. Cerebral palsy and developmental coordination disorder in children born preterm. Semin Fetal Neonatal Med. 2014;19:84–9. https://doi.org/10.1016/j.siny.2013.11.005
10. Pyhälä R, Hovi P, Lahti M, et al. Very low birth weight, infant growth, and autism-spectrum traits in adulthood. Pediatrics 2014;134:1075–83. https://doi.org/10.1542/peds.2014-1097
11. Franz AP, Bolat UG, Bolat H, et al. Attention-deficit/hyperactivity disorder and very preterm/very low birth weight: a meta-analysis. 2018;141(1):e20171645. doi: 10.1542/peds.2017-1645
12. Cairney J, Hay J, Faught B, et al. Developmental Coordination Disorder, Self-efficacy toward physical activity, and play: does gender matter? APAQ 2005;22:67-82. https://doi.org/10.1123/apaq.22.1.67
13. Brown DMY, Cairney J. The synergistic effect of poor motor coordination, gender and age on self-concept in children: A longitudinal analysis. Res Dev Disabil 2020;98:103576. https://doi.org/10.1016/j.ridd.2020.103576
14. Lingam R, Golding J, Jongmans MJ, et al. The association between developmental coordination disorder and other developmental traits. Pediatrics 2010;126(5), e1109–e1118. https://doi.org/10.1542/peds.2009-2789

15. Gomez A, Piazza M, Jobert A, et al. Mathematical difficulties in developmental coordination disorder: Symbolic and nonsymbolic number processing. Res Dev Disabil 2015;43–44:167–78. https://doi.org/10.1016/j.ridd.2015.06.011
16. Rasmussen P, Gillberg C. Natural outcome of ADHD with developmental coordination disorder at age 22 years: A controlled, longitudinal, community-based study. J Am Acad Child Adolesc Psychiatry 2000;39:1424–31. https://doi.org/10.1097/00004583-200011000-00017
17. Twilhaar ES, de Kieviet JF, Aarnoudse-Moens CSH, et al. Academic performance of children born preterm: A meta-analysis and meta-regression. Arch Dis Child Fetal Neonatal Ed 2018;103:F322-F330. https://doi.org/10.1136/archdischild-2017-312916
18. Jangmo A, Stålhandske A, Chang Z, et al. Attention-deficit/hyperactivity disorder, school performance, and effect of medication. J Am Acad Child Adolesc Psychiatry 2019;58(4):423-32. https://doi.org/10.1016/j.jaac.2018.11.014
19. The Swedish National Agency for Education. Analyser av familjebakgrundens betydelse för skolresultaten och skillnader mellan skolor. 2018. Retrieved from: https://www.skolverket.se/publikationsserier/rapporter/2018/analyser-av-familjebakgrundens-betydelse-for-skolresultaten-och-skillnader-mellan-skolor?sv.target=12.3253018117f029df83d13e&sv.12.3253018117f029df83d13e.route=/settings. Accessed June 13, 2022.
20. The Swedish National Agency for Education. Overview of the Swedish upper secondary school. 2012. Retrieved from: https://www.skolverket.se/download/18.6bfaca41169863e6a659a16/1553964183391/pdf2748.pdf. Accessed June 13, 2022.
21. Swedish Public Employment Service. 2016. Perspektiv på ungdomsarbetslösheten. Retrieved from: https://arbetsformedlingen.se/download/18.3e623d4f16735f3976ea8e/Perspektiv%20p%C3%A5%20ungdomsarbetsl%C3%B6sheten.pdf. Accessed June 13, 2022.
22. Statistics Sweden. Labour Force Surveys, Grundtabeller årsmedeltal 2020. Retrieved from: https://www.scb.se/hitta-statistik/statistik-efter-amne/arbetsmarknad/arbetskraftsundersokningar/arbetskraftsundersokningarna-aku/pong/tabell-och-diagram/icke-sasongrensade-data/grundtabeller-aku-1574-ar-ar/2020. Accessed June 13, 2022.
23. The Delegation for the Employment of Young People and Newly Arrived Migrants. 2016. Fullföljd gymnasieutbildning. Retrieved from: https://www.dua.se/Fullfoljd-Gymnasieutbildning. Accessed June 13, 2022.
24. Hultkrantz L, Karpaty P, Vimefall E. Education-earnings linkage for assessing societal benefits of interventions for children and youth in Sweden. Psychosocial Intervention 2017;26:171–9. https://doi.org/10.1016/j.psi.2017.06.001
25. Statistics Sweden. Population 16-74 years of age by region, highest level of education, age and sex. Year 1985 - 2020. Retrieved from: https://www.statistikdatabasen.scb.se/pxweb/sv/ssd/START__UF__UF0506/Utbildning/?loadedQueryId=57012&timeType=from&timeValue=2000. Accessed June 13, 2022 2021.
26. Swedish Higher Education Authority. Higher education in Sweden - 2018 status report. 2018. Retrieved from: https://english.uka.se/about-us/publications/reports--guidelines/reports--guidelines/2018-06-29-higher-education-in-sweden---2018-status-report.html. Accessed June 13, 2022.

For peer review only

27. Persson K, Strömberg B. Structured observation of motor performance (SOMP-I) applied to preterm and full term infants who needed neonatal intensive care. Across-sectional analysis of progress and quality of motor performance at ages 0-10 months. Early Hum Dev 1995;43(3):205–24. https://doi.org/10.1016/0378-3782(95)01678-3
28. Montgomery C, Johansen K, Lucas S, et al. The Structured Observation of Motor Performance in Infants can detect cerebral palsy early in neonatal intensive care recipients. Early Hum Dev 2017;113:31–9. https://doi.org/10.1016/j.earlhumdev.2017.07.009
29. Hemgren E, Persson K. Deficits in motor co-ordination and attention at 3 years of age predict motor deviations in 6.5-year-old children who needed neonatal intensive care. Child Care Health Dev 2009;35:120–9. https://doi.org/10.1111/j.1365-2214.2008.00896.x
30. Stott DH, Moyes EA, Henderson SE. Test of Motor Impairment, Henderson Revision. Brook Educational Publishing. 1984.
31. Holle B, Bönnelycke K, Kemp E, et al. Motorisk-perceptuell utveckling. Psykologiförlaget AB. 1977.
32. von Elm E, Altman DG, Egger M, et al. The strengthening the reporting of observational studies in epidemiology (STROBE) statement: guidelines for reporting observational studies. Int J Surg 2014;12:1495–9. doi: 10.1016/j.ijsu.2014.07.013.
33. Statistics Sweden. Longitudinal integrated database for health insurance and labour market studies (LISA). 2022. Retrieved from: https://www.scb.se/en/services/ordering-data-and-statistics/ordering-microdata/vilka-mikrodata-finns/longitudinella-register/longitudinal-integrated-database-for-health-insurance-and-labour-market-studies-lisa/. Accessed June 13, 2022.
34. National Board of Health and Welfare. National Patient Register. 2022. Retrieved from: https://www.socialstyrelsen.se/en/statistics-and-data/registers/national-patient-register/. Accessed June 13, 2022.
35. Ludvigsson JF, Svedberg P, Olén O, et al. The longitudinal integrated database for health insurance and labour market studies (LISA) and its use in medical research. Eur J Epidemiol 2019;34:423-37. https://doi.org/10.1007/s10654-019-00511-8
36. Allotey J, Zamora J, Cheong-See F, et al. Cognitive, motor, behavioural and academic performances of children born preterm: a meta-analysis and systematic review involving 64 061 children. BJOG. 2018;125:16–25. https://doi.org/10.1111/1471-0528.14832
37. Licari MK, Alvares GA, Bernie C, et al. The unmet clinical needs of children with developmental coordination disorder. Pediatr Res 2021;90:826–31. https://doi.org/10.1038/s41390-021-01373-1
38. Hunt J, Zwicker JG, Godecke E, et al. Awareness and knowledge of developmental coordination disorder: A survey of caregivers, teachers, allied health professionals and medical professionals in Australia. Child Care Health Dev 2021;47:174–83. https://doi.org/10.1111/cch.12824
39. Tamplain P, Miller HL. What Can We Do to Promote Mental Health Among Individuals With Developmental Coordination Disorder? Curr Dev Disord Rep. 2021;8:24–31. https://doi.org/10.1007/s40474-020-00209-7.
40. Doyle LW, Spittle A, Anderson PJ, et al. School-aged neurodevelopmental outcomes for children born extremely preterm. Arch Dis Child. 2021;106:834–38. https://doi.org/10.1136/archdischild-2021-321668.

For peer review only

41. Raju TNK, Buist AS, Blaisdell CJ, et al. Adults born preterm: a review of general health and system-specific outcomes. Acta Paediatr 2017;106:1409–37. https://doi.org/10.1111/apa.13880
42. O'Nions E, Wolke D, Johnson S, et al. Preterm birth: Educational and mental health outcomes. Clin Child Psychol Psychiatry. 2021;26:750–9. https://doi.org/10.1177/13591045211006754
43. Twilhaar ES, de Kieviet JF, van Elburg RM, et al. Academic trajectories of very preterm born children at school age. Arch Dis Child Fetal Neonatal Ed 2019;104:F419–23. https://doi.org/10.1136/archdischild-2018-315028
44. Blencowe H, Cousens S, Narwal R, et al. Articles National, regional, and worldwide estimates of preterm birth rates in the year 2010 with time trends since 1990 for selected countries: a systematic analysis and implications. Lancet 2012;379(9832):2162-72. https://doi.org/10.1016/S0140-6736(12)60820-4
45. Spittle AJ, Thompson DK, Olsen JE, et al. Predictors of long-term neurodevelopmental outcomes of children born extremely preterm. Semin Perinatol 2021;45:151482. https://doi.org/10.1016/j.semperi.2021.151482
46. Hutchinson EA, de Luca CR, Doyle LW, et al. School-age outcomes of extremely preterm or extremely low birth weight children. Pediatrics 2013;131(4):e1053–e1061. https://doi.org/10.1542/peds.2012-2311
47. Marlow N, Ni Y, Lancaster R, et al. No change in neurodevelopment at 11 years after extremely preterm birth. Arch Dis Child Fetal Neonatal Ed 2021;106(4):418-24. https://doi.org/10.1136/archdischild-2020-320650
48. Morsing E, Lundgren P, Hård AL, et al. Neurodevelopmental disorders and somatic diagnoses in a national cohort of children born before 24 weeks of gestation. Acta Paediatr 2022;111(6):1167–75. https://doi.org/10.1111/apa.16316

For peer review only

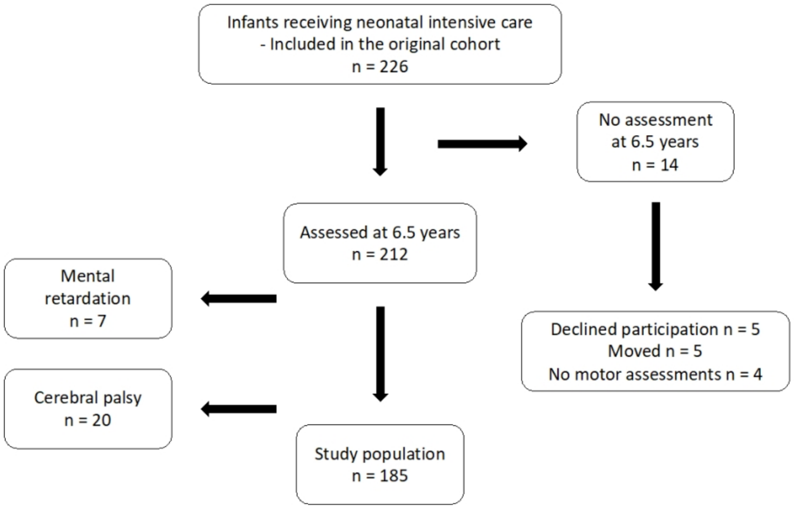

Figure 1. Flow-chart of recruitment.

720x405mm (47 x 47 DPI)

Supplementary file 1. ICD-8/9 codes included in the variable "Diagnoses posing a risk of motor problems".

ICD-code	Diagnosis
040–046	Poliomyelitis and other enterovirus diseases of central nervous system (ICD-8)
190–199	Malignant neoplasm of other and unspecified sites (ICD-8 and ICD-9)
210–228	Benign neoplasms (ICD-8)
300–316	Neurotic disorders, personality disorders, and other nonpsychotic mental disorders (ICD-8)
320–324	Inflammatory diseases of central nervous system (ICD-8)
330–337	Hereditary and degenerative diseases of the central nervous system (ICD-9)
340–349	Other diseases of central nervous system (ICD-8)
430–438	Cerebrovascular disease (ICD-8 and ICD-9)
710–719	Arthropathies and related disorders (ICD-9)
740–759	Congenital anomalies (ICD-8 and ICD-9)
760–779	Certain conditions originating in the perinatal period (ICD-9) or certain causes of perinatal morbidity and mortality (ICD-8)
780–789	Symptoms referable to systems or organs (ICD-8)
850–854	Intracranial injury, excluding those with skull fracture (ICD-9)

Note: ICD = International Statistical Classification of Diseases and Related Health Problems - Eight/Ninth revision

For peer review only

Supplementary file 2. Number of neurodevelopmental disorders registered in specialized outpatient care.

**ICD-10 Codes**		n (%)
F80-F89	Disorders of psychological development	2 (1.1)
F90-F98	Behavioral and emotional disorders with onset usually occurring in childhood and adolescence	10 (5.5)
R47-R49	Symptoms and signs involving speech and voice	2 (1.1)

F80-F89 includes: Developmental Coordination Disorder, Developmental language disorder, Dyslexia, Autism Spectrum Disorders and impairments in reading, writing and arithmetic.

F90-F98 includes: Attention Deficit Hyperactivity Disorder and Tic disorders.

R47-R49 includes: Dyslexia.

Note: ICD=International Statistical Classification of Diseases and Related Health Problems.

For peer review only

Reporting checklist for cohort study.

Based on the STROBE cohort guidelines.

Instructions to authors

Complete this checklist by entering the page numbers from your manuscript where readers will find each of the items listed below.

Your article may not currently address all the items on the checklist. Please modify your text to include the missing information. If you are certain that an item does not apply, please write "n/a" and provide a short explanation.

Upload your completed checklist as an extra file when you submit to a journal.

In your methods section, say that you used the STROBE cohortreporting guidelines, and cite them as:

von Elm E, Altman DG, Egger M, Pocock SJ, Gotzsche PC, Vandenbroucke JP. The Strengthening the Reporting of Observational Studies in Epidemiology (STROBE) Statement: guidelines for reporting observational studies.

For peer review only

		Reporting Item	Page Number
**Title and abstract**			
Title	#1a	Indicate the study's design with a commonly used term in the title or the abstract	1

Abstract	#1b	Provide in the abstract an informative and balanced summary of what was done and what was found	2
**Introduction**			
Background / rationale	#2	Explain the scientific background and rationale for the investigation being reported	4
Objectives	#3	State specific objectives, including any prespecified hypotheses	5
**Methods**			
Study design	#4	Present key elements of study design early in the paper	5
Setting	#5	Describe the setting, locations, and relevant dates, including periods of recruitment, exposure, follow-up, and data collection	5-6
Eligibility criteria	#6a	Give the eligibility criteria, and the sources and methods of selection of participants. Describe methods of follow-up.	5-6
Eligibility criteria	#6b	For matched studies, give matching criteria and number of exposed and unexposed	n/a
Variables	#7	Clearly define all outcomes, exposures, predictors, potential confounders, and effect modifiers. Give diagnostic criteria, if applicable	6-7
Data sources / measurement	#8	For each variable of interest give sources of data and details of methods of assessment (measurement). Describe comparability of assessment methods if there is more than	6

For peer review only

		one group. Give information separately for for exposed and unexposed groups if applicable.	
Bias	#9	Describe any efforts to address potential sources of bias	7
Study size	#10	Explain how the study size was arrived at	5
Quantitative variables	#11	Explain how quantitative variables were handled in the analyses. If applicable, describe which groupings were chosen, and why	7
Statistical methods	#12a	Describe all statistical methods, including those used to control for confounding	7
Statistical methods	#12b	Describe any methods used to examine subgroups and interactions	7
Statistical methods	#12c	Explain how missing data were addressed	7
Statistical methods	#12d	If applicable, explain how loss to follow-up was addressed	n/a
Statistical methods	#12e	Describe any sensitivity analyses	n/a

For peer review only

Results

Participants	#13a	Report numbers of individuals at each stage of study—eg numbers potentially eligible, examined for eligibility, confirmed eligible, included in the study, completing follow-up, and analysed. Give information separately for for exposed and unexposed groups if applicable.	5
Participants	#13b	Give reasons for non-participation at each stage	5
Participants	#13c	Consider use of a flow diagram	6 and figure 1
Descriptive data	#14a	Give characteristics of study participants (eg demographic, clinical, social) and information on exposures and potential confounders. Give information separately for exposed and unexposed groups if applicable.	8
Descriptive data	#14b	Indicate number of participants with missing data for each variable of interest	Page 7, and table 2, 4 and 5
Descriptive data	#14c	Summarise follow-up time (eg, average and total amount)	5-6
Outcome data	#15	Report numbers of outcome events or summary measures over time. Give information separately for exposed and unexposed groups if applicable.	9 and table 2-3

For peer review only

Main results	#16a	Give unadjusted estimates and, if applicable, confounder-adjusted estimates and their precision (eg, 95% confidence interval). Make clear which confounders were adjusted for and why they were included	Table 3
Main results	#16b	Report category boundaries when continuous variables were categorized	Table 1-5
Main results	#16c	If relevant, consider translating estimates of relative risk into absolute risk for a meaningful time period	n/a
Other analyses	#17	Report other analyses done—eg analyses of subgroups and interactions, and sensitivity analyses	12-13 and table 4-5
**Discussion**			
Key results	#18	Summarise key results with reference to study objectives	14
Limitations	#19	Discuss limitations of the study, taking into account sources of potential bias or imprecision. Discuss both direction and magnitude of any potential bias.	16
Interpretation	#20	Give a cautious overall interpretation considering objectives, limitations, multiplicity of analyses, results from similar studies, and other relevant evidence.	16-17
Generalisability	#21	Discuss the generalisability (external validity) of the study results	16-17
**Other Information**			

For peer review only

Funding	#22	Give the source of funding and the role of the funders for the present study and, if applicable, for the original study on which the present article is based	18

Notes:

- 14b: 7, and table 2, 4 and 5 The STROBE checklist is distributed under the terms of the Creative Commons Attribution License CC-BY. This checklist was completed on 28. December 2022 using https://www.goodreports.org/, a tool made by the EQUATOR Network in collaboration with Penelope.ai

For peer review only

BMJ Open

The impact of developmental coordination disorder in childhood on educational outcomes in adulthood among neonatal intensive care recipients: a register-based longitudinal cohort study

Journal:	*BMJ Open*
Manuscript ID	bmjopen-2023-071563.R1
Article Type:	Original research
Date Submitted by the Author:	16-May-2023
Complete List of Authors:	Persson, Isak; Uppsala University, Department of Women's and Children's Health<br>Sampaio, Filipa; Uppsala University, Department of Public Health and Caring Sciences<br>Samkharadze, Tengiz; Uppsala University, Department of Public Health and Caring Sciences<br>Ssegonja, Richard; Uppsala University, Department of Public Health and Caring Sciences<br>Johansen, Kine; Uppsala University, Department of Women's and Children's Health
**Primary Subject Heading**:	Paediatrics
Secondary Subject Heading:	Neurology
Keywords:	NEONATOLOGY, Developmental neurology & neurodisability < PAEDIATRICS, REGISTRIES, Neonatal intensive & critical care < INTENSIVE & CRITICAL CARE

SCHOLARONE™
Manuscripts

I, the Submitting Author has the right to grant and does grant on behalf of all authors of the Work (as defined in the below author licence), an exclusive licence and/or a non-exclusive licence for contributions from authors who are: i) UK Crown employees; ii) where BMJ has agreed a CC-BY licence shall apply, and/or iii) in accordance with the terms applicable for US Federal Government officers or employees acting as part of their official duties; on a worldwide, perpetual, irrevocable, royalty-free basis to BMJ Publishing Group Ltd ("BMJ") its licensees and where the relevant Journal is co-owned by BMJ to the co-owners of the Journal, to publish the Work in this journal and any other BMJ products and to exploit all rights, as set out in our licence.

The Submitting Author accepts and understands that any supply made under these terms is made by BMJ to the Submitting Author unless you are acting as an employee on behalf of your employer or a postgraduate student of an affiliated institution which is paying any applicable article publishing charge ("APC") for Open Access articles. Where the Submitting Author wishes to make the Work available on an Open Access basis (and intends to pay the relevant APC), the terms of reuse of such Open Access shall be governed by a Creative Commons licence – details of these licences and which Creative Commons licence will apply to this Work are set out in our licence referred to above.

Other than as permitted in any relevant BMJ Author's Self Archiving Policies, I confirm this Work has not been accepted for publication elsewhere, is not being considered for publication elsewhere and does not duplicate material already published. I confirm all authors consent to publication of this Work and authorise the granting of this licence.

For peer review only

The impact of developmental coordination disorder in childhood on educational outcomes in adulthood among neonatal intensive care recipients: a register-based longitudinal cohort study

Isak Persson[a], Filipa Sampaio[b], Tengiz Samkharadze[b], Richard Ssegonja[b,c], Kine Johansen[a]

[a] Department of Women's and Children's Health, Uppsala University, Uppsala University Children's Hospital, Uppsala, Sweden
[b] Department of Public Health and Caring Sciences, Uppsala University, Uppsala, Sweden
[c] Department of Medical Sciences, Respiratory-, Allergy- and Sleep Medicine Research Unite, Uppsala University, Uppsala University Hospital, Uppsala, Sweden

Corresponding author
Kine Johansen (ORCID 0000-0002-9212-8452), Department of Women's and Children's Health, Uppsala University, Uppsala University Hospital, 751 85 Uppsala, Sweden. Tel +46 73 904 29 94, e-mail: kine.johansen@kbh.uu.se.

Word count: 4551

Number of tables: 5

Number of figures: 0

For peer review only

ABSTRACT

Objectives: Developmental Coordination Disorder (DCD) is related to poorer educational outcomes among children and adolescents. Evidence on whether this association persists into adulthood is lacking. To investigate whether childhood DCD affected educational outcomes among adults, and whether this was affected by sex or a co-occurring attention deficit.

Design: Register-based longitudinal cohort study.

Setting: Neonatal intensive care (NIC) recipients born at Uppsala University Children's Hospital, Uppsala, Sweden, followed through the age of 28 years.

Participants: 185 NIC recipients born 1986-1989 were included.

Primary and secondary outcome measures: At 6.5 years of age, 46 (24.6%) were diagnosed with probable DCD (pDCD). Using register-based longitudinal data, we compared those with pDCD to those without in terms of: i) age at graduation from Upper Secondary School (USS), and ii) highest completed level of education at 28 years of age.

Results: The median age at USS graduation was 19 years, with similar graduation ages and ranges between the pDCD and non-pDCD groups. At 28 years, 33% of participants had a bachelor's or master's degree, with no significant difference between the groups. However, the proportion that had attained a degree was higher among those without DCD, and women without DCD attained the highest level of education. Educational outcomes were similar for those with pDCD, regardless of whether they had a co-occurring attention deficit.

Conclusions: pDCD during childhood may have lasting impact on educational outcomes, particularly among women with pDCD, emphasizing the need for intervention and support to optimize learning and development. Raising awareness of DCD among parents, healthcare and educational professionals may enable early identification and tailored interventions for children with DCD in schools throughout their academic careers, which can mitigate negative consequences associated with pDCD.

Keywords

Developmental neurology & neurodisability, neonatal intensive & critical care, neonatology, registries

For peer review only

Strengths and limitations

- Strengths of this study include the longitudinal design and the use of national registers with high coverage and a low rate of missing data.
- It is one of few papers that follows educational outcomes in children with pDCD into adulthood, and the only one of these focusing on DCD specifically.
- Educational outcomes were measured solely as a binary outcome, without considering academic performance or any support received to graduate.
- Our analyses would have benefitted of a population-matched control group and measures of participants' functioning in adulthood for validation.
- The special characteristics of the study sample and the small sample size, could potentially limit the generalizability of the results and the ability to detect true differences, especially in subgroup analyses.

For peer review only

INTRODUCTION

Developmental Coordination Disorder (DCD) is a neurodevelopmental disorder that affects motor skills, which significantly interferes with daily activities, such as play, school-related and leisure activities [1]. With a prevalence of 5-6% among children [1], DCD affects approximately one in every 20 school-aged children. Moreover, it frequently co-occurs with other neurodevelopmental disorders, such as attention deficit hyperactivity disorder (ADHD), dyslexia, dyscalculia, developmental language disorder, and autism spectrum disorder [1,2]. While DCD once was considered a childhood disorder, research suggests that it is a lifelong condition [1]. Knowing that DCD affect school-related activities it is important to know if the disorder affects educational outcomes, as this closely correlates with both employment opportunities and higher incomes [3].

Children with DCD are more likely to have lower educational outcomes in childhood and adolescence [4–7], experience difficulties with language skills [8], impaired numerical cognition abilities [9], and executive dysfunction compared to typically developing peers [1,9]. Furthermore, DCD is also known to affect handwriting [1], which can hinder academic success. Harowell et al. reported lower national exams grades for 16-year-old English adolescents with DCD compared to their peers without DCD [5]. In addition, de Waal et al. found that girls with DCD achieved better outcomes than boys with DCD [10].

However, educational outcomes in children with DCD can be affected by several other factors. For example, prematurity and ADHD are well-known to be associated with poorer educational outcomes in childhood and adolescence [11–14]. That premature born children are at increased risk of neurodevelopmental disorders [15], and that neurodevelopmental disorders commonly co-occur further complicates the picture [2]. In fact, studies suggest that individuals with ADHD and a co-occurring DCD tend to have poorer educational outcomes than those diagnosed with ADHD alone [6,7,16]. Other factors, such as socioeconomic background, parental educational level, migration background, and income can also affect educational outcomes [17]. Moreover, DCD may affect boys and girls differently. For example, one a study by Brown and Cairney found that girls with poor motor coordination had lower self-perceptions of academic competence, athletic ability, and physical appearance compared to boys with DCD [18], and these differences became more pronounced with age.

In Sweden, education is tax-financed and offered free-of-charge. After nine years of compulsory schooling, i.e. at approximately 15 years of age, the majority of the adolescents choose to start Upper Secondary School (USS), an elective preparatory or vocational program lasting for three years [19]. Graduation from USS is a prerequisite for all higher education and

For peer review only

is commonly requested in the labour market for employment, even for occupations where educational requirements have traditionally been low [20]. In 2021, 31.4% of the Swedish labour force without graduation from USS were unemployed, compared to 7.8% among those who had graduated from USS without completing any post-secondary education [21]. According to the Delegation for the Employment of Young People and Newly Arrived Migrants, 65% of all students born in 1989 graduated from USS before age 20 and 91% before age 25 [22]. There was also a considerable gender difference; at 25 years of age, 12% of men lacked graduation compared to 6% of women [22]. The societal costs of one individual not finishing USS in Sweden is estimated at 163,000 € [23]. In 2021, 30% of the population aged 25-64 had attained at least a bachelor's degree [24], and the average age to reach this level was 28 years [3]. At age 40, 39% of women and 26% of men had attained at least a bachelor's degree [25].

Although poorer educational outcomes of individuals with DCD is well-documented in childhood and adolescence, there is a paucity of studies examining if this association persists into adulthood. Therefore, using a register-based longitudinal design, we explored, using a cohort of Swedish neonatal intensive care (NIC) recipients, how meeting the diagnostic criteria for DCD in childhood, affected educational outcomes as adults. Additionally, we investigated whether sex or a co-occurring attention deficit affected educational outcomes.

MATERIAL AND METHODS

Study design and participants

In this register-based longitudinal cohort study, we used data from a cohort of NIC recipients born at Uppsala University Children's Hospital, Sweden, between 1986 and 1989. All surviving infants (n=246) treated at the NIC unit during this period, who were residents in Uppsala County, were enrolled in a longitudinal follow-up study [26]. Inclusion criteria for NIC were: 1) respiratory therapy or treatment with continuous positive airway pressure, 2) total parenteral nutrition, 3) continuous intravenous treatment of neonatal convulsions, 4) severe asphyxia (Apgar score ≤ 4 at 5 min), and 5) all very preterm infants (< 32 completed gestational weeks (GW)) [26].

The children participated in repeated assessments from estimated date of birth up to 10 years of age [27,28]. At 6.5 years, 212 children were assessed using the Standardized Test of Motor Impairment (TOMI) [29], the predecessor of the Movement ABC [30], the Motor-Perceptual Development (MPU) [31], and the Combined Assessment of Motor Performance and Behaviour (CAMPB) [27,32] (Supplementary file 1). TOMI evaluates children's motor

For peer review only

abilities, specifically manual dexterity, ball skills, and balance [29], while MPU assesses gross motor skills, eye-hand coordination, perception, activities of daily living, and academic skills [31]. The CAMPB model, developed during the original cohort study, was used to evaluate the children's coordination and attention [32]. Based on this assessment, the children were retrospectively classified into motor deviation categories [27], including DCD defined according to the Diagnostic and Statistical Manual of Mental Disorders Text Revision Fourth Edition (DSM-IV-TR) (n = 46) [33]. For the purpose of the present study, those diagnosed with cerebral palsy (n = 20) and mental retardation (n = 7) were excluded. This left us with 185 participants eligible for participation. During the initial study period, Uppsala County had 280 000 inhabitants with a slightly younger and slightly more highly educated population than the national average [28].

This study adhered to the STROBE cohort checklist recommendations [34].

Data sources

We collected data on sex, gestational age, and outcome of the motor assessment at 6.5 years from the original cohort study. In 2019, data on educational outcomes was retrieved from the 'Longitudinal Integrated Database for Health Insurance and Labour Market Studies' (LISA) [35] and linked to data on diagnoses and healthcare consumption from the 'National Patient Register' [36] and the 'Cause of Death Register' [37]. Linkage of registers was possible due to the personal identity numbers allocated to all Swedish residents. The National Board of Health and Welfare matched and anonymized the data before delivering it to the last author. Data were available for the years 2002-2018.

Outcome variables

The LISA-register integrates yearly information about education, employment and demographic characteristics. Education level is reported on an ordinal scale ranging from less than 9 years compulsory school to a licentiate's or doctor's degree [38]. We used this variable to create several derived variables, including a continuous variable indicating the age at which the participants graduated from USS based on the year of graduation reported in the register. Additionally, we created three categorical variables indicating whether participants had graduated from USS by age 19, 24 and 29 (yes/no), as well as a categorical variable indicating their educational level at age 28. We constructed age-specific binary variables for graduation from USS at 19 and 24 years of age based on data on average graduation rates in the Swedish population born in 1989 [22], while 29 years was the final point of measurement that allowed for analysis of the entire sample. We chose to investigate educational levels based on the

mean age for attaining a bachelor's degree in Sweden, which is 28 years [3]. Additionally, we created a binary variable indicating whether participants had graduated from USS by the end of the study.

Measures

The participants were categorized into two groups based on whether or not they met the diagnostic criteria for *DCD* at 6.5 years of age [27]: i.e. those without motor deviations, motor delays, or motor problems were grouped together as "No DCD", and compared to those who met the DCD criteria according to DSM-IV-TR, named "probable DCD" (pDCD). *Sex* was analysed as a binary measure (men/women). Participants assessed with a minor or pronounced attention deficit according to CAMPB at 6.5 years were defined as having a co-occurring attention deficit in childhood [27]. *Gestational age* was categorized as extremely preterm (22–27 GW), very preterm (28–32 GW), moderately preterm (32–36 GW), or full-term (37–42 GW). Diagnose codes obtained from the Swedish National Patient Register were grouped according to the International Statistical Classification of Diseases and Related Health Problems - eighth/ninth revision (ICD-8/9), but not specified in detail. Therefore, all information about diagnosis of neurodevelopmental disorders was combined into one variable indicating whether or not the participants had any *neurodevelopmental disorder* (yes/no). We categorized the diagnosis groups recorded during participants' first year as either posing an increased risk of motor problems or not. The diagnoses that posed an increased risk were summed into a continuous variable representing the total *number of diagnoses*. Please see supplementary file 2 and 3 for a detailed list of ICD-codes. *Hospital days* describe the total number of days the participants spent in inpatient care during their first year of life.

Missing data

Two women with pDCD missed all educational data in the LISA-register and were excluded from all statistical analyses, leaving a total sample of 183 participants. Additionally, two other women with pDCD had incomplete register data. Data from these participants was used when values could be assigned to the categorical outcome variables indicating graduation from USS, but were otherwise excluded. When analysing level of education at 28 years of age, four participants who had emigrated were lost to follow-up.

Nine participants had missing data on diagnoses and hospital days during the first year of life. To minimize loss of data when performing the regression analysis, we imputed the data using multiple imputation by chained equations. Five datasets were imputed using all available variables as predictors.

For peer review only

Statistical analysis

Descriptive statistics were used to characterize our sample and are presented as numbers and percentages. We compared participants with pDCD to those with No DCD. Additionally, we conducted sub-group analyses to explore educational outcomes based on sex or the presence of a co-occurring attention deficit in participants with pDCD. Differences in group characteristics were analysed using the Mann-Whitney U test, Chi-square test, or Fischer's exact test. Effect sizes were calculated using Hedges *g* and Cohen's *d,* and should be interpreted as small (≥0.20), medium (≥0.50), and large (≥0.80) effects [39]. Logistic regressions were conducted to explore the relationship between pDCD at 6.5 years of age (categorical) and having graduated from USS at 19, 24 or 29 years of age (categorical). The model was adjusted for sex (categorical), gestational age (categorical), any diagnosis of a neurodevelopmental disorder (categorical), the number of diagnoses posing a risk of motor problems (continuous), and hospital days (continuous) during the first year of life. A two-tailed p-value of <0.05 was considered statistically significant. Effect sizes were calculated using Social Science Statistics (https://www.socscistatistics.com/effectsize/default3.aspx), while all other analyses were performed using IBM SPSS Statistics 28 for Windows (SPSS Inc., Chicago, IL, USA).

Patient and public involvement

No patient or public involvement.

Ethical approval

Approval was given by the Swedish Ethical Review Authority (reg. no. 2019-04119). The register data were delivered anonymized but linked to the participants via unique identification numbers administered by the Swedish Board of Health and Welfare. All data were stored at the Department of Women's and Children's Health, Uppsala University, and on password protected servers held by the University. Data were handled in accordance to the General Data Protection Regulation and Swedish legislation. The processing of personal data was registered in Uppsala University's register of personal data processing and monitored by the Data Protection Officer.

RESULTS

Sample characteristics

Of the 183 participants, slightly more than half were men (n = 108, 59.0%) (Table 1). The majority were born moderate to late preterm (n = 70, 38.3%) or full-term (n = 62, 33.9%).

For peer review only

The corresponding numbers for the very and extremely preterm were 42 (23.0%) and 9 (4.9%), respectively. In total, 14 participants (7.7%) had a registered neurodevelopmental disorder recorded in specialized outpatient care (supplementary file 3). Of these, 8 (57.1%) fulfilled the diagnostic criteria for DCD at 6.5 years of age. The study sample had a median of four diagnoses that posed a risk of motor problems (interquartile range [IQR] 2.0-5.0) recorded during their first year of life, and they spent a median of 24 days in hospital during the same period (IQR 8.8-45.0).

Except that participants with pDCD had a higher rate of neurodevelopmental disorder in specialized outpatient care than those without DCD (p = .007), no other statistically significant group differences were found in sample characteristics. The pDCD-group contained slightly more men (n = 28, 62.2%) and had a median of 24 hospital days during their first year of life, which was two days longer than the median stay of 22 days for the No DCD-group. When comparing the distribution between gestational age groups, it can be noted that the pDCD-group had a higher relative count of extremely preterm births (n = 4, 8.9%) compared to those without DCD (n = 5, 3.6%), although not statistically significant (p = .502).

Table 1. Sample characteristics.

	Total	No DCD	pDCD	P	*g*
	n (%)<br>183 (100)	n (%)<br>138 (75.4)	n (%)<br>45 (24.6)		
**Sex**					
Men	108 (59.0)	80 (58.0)	28 (62.2)	.615	
Women	75 (41.0)	58 (42.0)	17 (37.8)		
**Gestational age**					
22–27 GW	9 (4.9)	5 (3.6)	4 (8.9)	.502	
28–31 GW	42 (23.0)	31 (22.5)	11 (24.4)		
32–36 GW	70 (38.3)	55 (39.9)	15 (33.3)		
37-42 GW	62 (33.9)	47 (34.1)	15 (33.3)		
**Neurodevelopmental disorder**					
Yes	14 (7.7)	6 (4.3)	8 (17.8)	.007	
No	169 (92.3)	132 (95.7)	37 (82.2)		
**Number of diagnoses posing a risk of motor problems**					
Median	4.0	4.0	4.0	.976	
Min-max	0.0–10.0	0.0–10.0	1.0–9.0		
IQR	2.0-5.0	2.0-5.0	2.0-5.0		
Mean (SD)	4.0 (1.9)	4.0 (1.9)	4.0 (1.9)		0.00
Missing	9.0 (4.9)	7.0 (5.1)	2.0 (4.4)		
**Number of hospital days**					
Median	24.0	23.5	26.0	.643	
Median	22.0	22.0	24.0		

For peer review only

Min-max	0.0–200.0	1.0–200.0	0.0–168.0	
IQR	8.8-45.0	8.0-43.0	9.0-53.0	
Mean (SD)	32.1 (30.5)	30.8 (29.2)	35.9 (34.1)	0.17
Missing	9 (4.9)	7 (5.1)	2 (4.4)	

Note: DCD=Diagnosed with Developmental Coordination Disorder; GW=gestational weeks, IQR=interquartile range, Min=minimum; Max=maximum; SD=Standard Deviation.

Educational outcomes

The median age at USS graduation for all participants was 19 years (Table 2). While the pDCD group graduated with a slightly wider spread (IQR 19.0-20.0 vs 19.0-19.0) (p = .022, g = 0.41), the graduation age and range were similar between the groups. At 19 years, 78.2% (n=108) of the No DCD-group and had 54.5% (n=24) of the pDCD-group had graduated (p = .002). At 24 years, the percentage of graduates was higher in the No-DCD group (92.8%, n=128) than in the pDCD-group (81.1%, n=36) (p = .044).

In total, 16 participants had not finished USS (8.7%) by the end of the study, seven of whom met the criteria for DCD (43.8%) at 6.5 years of age (p = .069). Regardless, the majority of participants (91.2%) had graduated from USS at 29 years. At 28 years, 33% of participants had attained a bachelor's or master's degree, with no statistically significant difference between the groups (p = .080). Nonetheless, the proportion of those without DCD with a bachelor's or master's degree (35.8%) was higher than those with pDCD (24.4%).

Table 2. Educational outcomes for participants who met the diagnostic criteria for DCD (pDCD) at 6.5 years of age and those who did not (No DCD).

	**Total**	**No DCD**	**pDCD**	**P**	***g***
	n (%)	n (%)	n (%)		
	183 (100)	138 (75.4)	45 (24.6)		
**Age (yrs) at graduation from USS**					
Median	19.0	19.0	19.0	.022	
Min-max	19.0–26.0	19.0–26.0	19.0–26.0		
IQR	19.0-19.0	19.0-19.0	19.0-20.0		
Mean (SD)	19.3 (1.0)	19.2 (0.8)	19.6 (1.4)		0.41
Missing data, total	18 (9.8)	9 (6.5)	9 (20.0)		
No graduation	16 (8.7)	9 (6.5)	7 (15.6)	.069	
Absent/incomplete register data	2 (1.1)		2 (4.4)		
**USS graduation at 19 yrs**					
Yes	132 (72.5)	108 (78.3)	24 (54.5)	.002	
No	50 (27.5)	30 (21.7)	20 (45.5)		
Missing data	1 (0.5)		1 (2.2)		
**USS graduation at 24 yrs**					
Yes	164 (90.1)	128 (92.8)	36 (81.8)	.044	
No	18 (9.9)	10 (7.2)	8 (18.2)		
Missing data	1 (0.5)		1 (2.2)		

For peer review only

**USS graduation at 29 yrs**				
Yes	166 (91.2)	129 (93.5)	37 (84.1)	.069
No	16 (8.8)	9 (6.5)	7 (15.9)	
Missing data	1 (0.5)		1 (2.2)	
**Level of education at 28 yrs**				
Compulsory school, 9 yrs	10 (5.6)	7 (5.1)	3 (7.3)	.080
Upper secondary education, ≤2 yrs	6 (3.4)	2 (1.5)	4 (9.8)	
Upper secondary education, 3 yrs	74 (41.6)	55 (40.1)	19 (46.3)	
Post-secondary education, <3 yrs	29 (16.3)	24 (17.5)	5 (12.2)	
Bachelor's or master's degree	59 (33.1)	49 (35.8)	10 (24.4)	
Missing data	5 (2.7)	1 (0.7)	4 (8.9)	

Note: DCD=Developmental Coordination Disorder; IQR=interquartile range; Min=minimum; Max=maximum; SD=Standard Deviation; USS=Upper Secondary School, Yrs=Years.

The contribution of covariates in explaining changes in graduation from USS was small (Table 3). However, a pDCD diagnosis negatively impacted graduation at both 19 and 24 years of age, in both unadjusted and adjusted models. Those with pDCD at 6.5 years of age were less likely to graduate at 19 (OR: .333, p = .003; 95% CI: .16-.68) and 24 (OR: .352, p = .041; 95% CI: .13-.96) years of age, compared to those without DCD. Adjustment for sex, gestational age, any neurodevelopmental disorder, number of diagnoses posing a risk of motor problems, and the number of hospital days during the first year of life did not change the odds of graduation significantly (OR: .315-.345).

Table 3. Logistic regression results for prediction of graduation at 19, 24 and 29 years of age.

Covariates	R^2	B	OR	95% CI	P
**USS graduation at 19 yrs**					
**Unadjusted**	.069				
pDCD (pDCD vs no DCD*)		-1.099	.333	(0.16, 0.68)	.003
**Adjusted**	.124-.149**				
pDCD (DCD vs no DCD*)		-1.056	.348	(.16, .75)	.007
Sex (men vs women*)		-.173	.841	(.41, 1.73)	.637
Gestational age					
22–27 GW vs full-term*		1.533	4.634	(.56, 38.23)	.154
28–31 GW vs full- term*		.716	2.046	(.60, 7.03)	.256
32–36 GW vs full-term*		.613	1.846	(.73, 4.66)	.194
Neurodevelopmental disorder		-.806	.447	(.13, 1.50)	.193
Number of diagnoses posing a risk of motor problems		-.211	.810	(.64, 1.02)	.074
Number of hospital days		-.007	.993	(.98, 1.01)	.467
**USS graduation at 24 yrs**					

For peer review only

**Unadjusted**	.045				
pDCD (pDCD vs no DCD*)		-1.045	.352	(0.13, 0.96)	.041
**Adjusted**	.089-.102**				
DCD (DCD vs no DCD*)		-1.106	.331	(.11, .96)	.041
Sex (men vs women*)		.109	1.115	(.39, 3.15)	.838
Gestational age					
22–27 GW vs full-term*		19.464	284 x 10^6	(.00, .00)	.999
28–31 GW vs full- term*		-.271	.763	(.13, 4.41)	.762
32–36 GW vs full-term*		.013	1.013	(.26, 3.99)	.985
Neurodevelopmental disorder		-.195	.823	(.15, 4.58)	.824
Number of diagnoses posing a risk of motor problems		-.144	.866	(0.63, 1.19)	.371
Number of hospital days		.001	1.001	(0.98, 1.03)	.925
**USS graduation at 29 yrs**					
**Unadjusted**	.040				
pDCD (pDCD vs no DCD*)		-.998	.369	(0.13, 1.06)	.063
**Adjusted**	.079-.090**				
pDCD (pDCD vs no DCD*)		-1.007	.365	(.12, 1.11)	.076
Sex (men vs women*)		.033	1.033	(.34, 3.11)	.953
Gestational age					
22–27 GW vs full-term*		18.979	175 x 10^6	(.00, .00)	.999
28–31 GW vs full- term*		-.365	.694	(.10, 4.85)	.713
32–36 GW vs full-term*		.100	1.106	(.26, 4.89)	.892
Neurodevelopmental disorder		-.407	.666	(.12, 3.72)	.643
Number of diagnoses posing a risk of motor problems		-.152	.859	(.62, 1.19)	.360
Number of hospital days		.009	1.009	(.98, 1.04)	.579

Note: CI=Confidence Interval; DCD=Developmental Coordination Disorder; GW=gestational weeks; OR = Odds Ratio; R^2 = Neglekerke's R^2; yrs=years.
*Reference ** Range denotes the variance of the value of R^2 between different imputations

Educational outcomes by sex

There were no statistically significant differences in educational outcomes between men and women in the total sample or between those with pDCD and No DCD (Supplementary file 4). However, women with pDCD (n = 8, 50.0%) had a significantly lower proportion of USS graduates at age 19 (p = .017, g = 0.85) compared to women without DCD (n = 48, 82.8%) (Table 4). While not statistically significant, there was a similar tendency among men (p = .075, g = 0.47). At age 28, fewer women with pDCD had attained a bachelor's or master's degree (n = 4, 28.6%) compared to women without DCD (n = 28, 48.3%). Men with or without pDCD had comparable performance to women with pDCD (n = 6, 22.2% vs n = 21, 26.6%).

For peer review only

Table 4. Educational outcomes for men and women with or without DCD.

	Men n=108				Women n=75			
	**No DCD** n (%) 80 (74.1)	**pDCD** n (%) 28 (25.9)	**P**	*g*	**No DCD** n (%) 58 (77.3)	**pDCD** n (%) 17 (22.7)	**P**	*g*
**Age (yrs) at graduation from USS**								
Median	19.0	19.0	.157		19.0	19.0	.067	
Min-max	19.0–22.0	19.0–26.0			19.0–26.0	19.0–24.0		
IQR	19.0-19.0	19.0-20.0			19.0-19.0	19.0-20.0		
Mean (SD)	19.2 (.6)	19.6 (1.4)		0.47	19.2 (1.0)	19.8 (1.5)		0.85
Missing data, total	6 (7.5)	4 (14.3)			3 (5.2)	5 (29.4)		
No graduation	6 (7.5)	4 (14.3)	.281		3 (5.2)	3 (17.7)	.111	
Absent/incomplete register data						2 (11.8)		
**USS graduation at 19 yrs**								
Yes	60 (75.0)	16 (57.1)	.075		48 (82.8)	8 (50.0)	.017	
No	20 (25.0)	12 (42.9)			10 (17.2)	8 (50.0)		
Missing data						1 (5.9)		
**USS graduation at 24 yrs**								
Yes	74 (92.5)	23 (82.1)	.148		54 (93.1)	13 (81.3)	.168	
No	6 (7.5)	5 (17.9)			4 (6.9)	3 (18.8)		
Missing data						1 (5.9)		
**USS graduation at 29 yrs**								
Yes	74 (92.5)	24 (85.7)	.281		55 (94.8)	13 (81.3)	.111	
No	6 (7.5)	4 (14.3)			3 (5.2)	3 (18.8)		
Missing data						1 (5.9)		
**Level of education at 28 yrs**								
Compulsory school, 9 yrs	4 (5.1)	2 (7.4)	.665		3 (5.2)	1 (7.1)	.082	
Upper secondary education, ≤2 yrs	2 (2.5)	2 (7.4)			0 (0)	2 (14.3)		
Upper secondary education, 3 yrs	38 (48.1)	14 (51.9)			17 (29.3)	5 (35.7)		
Post-secondary education, <3 yrs	14 (17.7)	3 (11.1)			10 (17.2)	2 (14.3)		
Bachelor's or master's degree	21 (26.6)	6 (22.2)			28 (48.3)	4 (28.6)		
Missing data	1 (1.3)	1 (3.6)				3 (17.6)		

Note: DCD=Developmental Coordination Disorder; IQR=interquartile range; Min=minimum; Max=maximum; SD=Standard Deviation; USS=Upper Secondary School, Yrs=Years.

Educational outcomes for pDCD and attention deficits

Half of the participants with pDCD had a co-occurring attention deficit according to CAMPB at 6.5 years of age (n = 22, 48.9%) (Table 5). Educational outcomes were similar between those with pDCD with or without an attention deficit, but fewer of those with pDCD and an attention deficit had graduated from USS at all ages. The differences were most evident at 19

For peer review only

(42.9% vs 65.2%; *p* = .137) and 24 (72.7% vs 90.9%; *p* = .240) years of age. At the age of 28, the percentage of participants with a degree from higher education was similar in both groups (23.8% vs 25.0%). However, among participants with pDCD and an attention deficit, 22.7% (n = 5) had not graduated from USS by the age of 29.

Table 5. Educational outcomes for participants with pDCD with or without a co-occurring attention deficit.

	pDCD	pDCD -	pDCD +	P	*d*
	n (%)	n (%)	n (%)		
	45 (100)	23 (51.1)	22 (48.9)		
**Age (yrs) at graduation from USS**					
Median	19.0	19.0	19.0	.320	
Min-max	19.0–26.0	19.0–24.0	19.0–26.0		
IQR	19.0-20.0	19.0-19.8	19.0-20.0		
Mean (SD)	19.6 (1.4)	19.5 (1.2)	19.9 (1.8)		0.26
Missing data, total	9 (20.0)	3 (13.0)	6 (27.3)		
No graduation	7 (15.6)	2 (8.7)	5 (22.7)	.412	
Absent/incomplete data	2 (4.4)	1 (4.3)	1 (4.6)		
**USS graduation at 19 yrs**					
Yes	24 (54.5)	15 (65.2)	9 (42.9)	.137	
No	20 (45.5)	8 (34.8)	12 (57.1)		
Missing data	1 (2.2)		1 (4.5)		
**USS graduation at 24 yrs**					
Yes	36 (81.8)	20 (90.9)	16 (72.7)	.240	
No	8 (18.2)	2 (9.1)	6 (27.3)		
Missing data	1 (2.2)	1 (4.3)			
**USS graduation at 29 yrs**					
Yes	37 (84.1)	20 (90.9)	17 (77.3)	.412	
No	7 (15.9)	2 (9.1)	5 (22.7)		
Missing data	1 (2.2)	1 (4.3)			
**Level of education at 28 yrs**					
Compulsory school, 9 yrs	3 (7.3)	1 (5.0)	2 (9.5)	.755	
Upper secondary education, ≤2 yrs	4 (9.8)	1 (5.0)	3 (14.3)		
Upper secondary education, 3 yrs	19 (46.3)	11 (55.0)	8 (38.1)		
Post-secondary education, <3 yrs	5 (12.2)	2 (10.0)	3 (14.3)		
Bachelor's or master's degree	10 (24.4)	5 (25.0)	5 (23.8)		
Missing data	4 (8.9)	3 (13.0)	1 (4.5)		

Note: DCD=Developmental Coordination Disorder; IQR=interquartile range; Min=minimum; Max=maximum; SD=Standard Deviation; USS=Upper Secondary School, Yrs=Years.

DISCUSSION

Using longitudinal individual level data from national registers on a cohort of NIC recipients, we found that their educational level was comparable to the national average at ages 19 and 24 [22] While fewer participants with pDCD had graduated from USS at these ages, the

For peer review only

variation was similar between groups and most had graduated by age 24. By age 28, 33.1% of participants had attained a bachelor's or master's degree, similar to the national average [24]. We observed that fewer women with pDCD had graduated from USS at 19 years of age. However, apart from this finding, no other significant differences in educational outcomes were observed between sexes or among those with pDCD, with or without a co-occurring attention deficit, according to CAMPB at 6.5 years of age.

DCD and educational outcomes

While previous research suggests that DCD can negatively affect educational outcomes in childhood and adolescence [4–7], our findings indicate that this effect is less pronounced in adulthood. However, in our sample of NIC recipients, those with pDCD were less likely to graduate from USS at 19 and 24 years than their peers, and pDCD predicted poorer educational outcomes independently of other factors affecting education, such as prematurity, sex, and neurodevelopmental disorders [14,22,25]. Additionally, although the difference in post-secondary education completion rates was not statistically significant, those with pDCD (24.4%) were less likely to attain a bachelor's or master's degree by the age of 28 compared to those without DCD (35.8%). In Sweden, 30% of the general population (aged 25-64) attains a bachelor's degree on average at age 28 [3,24].

Our study suggests that adults with pDCD in childhood have positive educational outcomes based on graduation rates from USS. However, it is important to note that we did not consider academic performance or the need for special support, adjustments and accommodations in school. Previous research has shown that DCD can have a negative impact on exam results and that students with motor difficulties may not receive adequate support in school [5,40]. Similarly, Yngve et al. reported that despite the experienced need for support, half of students with special education needs did not receive accommodations in USS [40]. Furthermore, interviews with participants from our cohort who had motor difficulties at 6.5 years of age found that few participants recalled receiving any school-based support for their motor difficulties [41], instead credited their parents for their success.

Despite being a relatively new diagnosis when our participants started school, DCD remains unfamiliar to many [42,43]. According to parents, lack of teacher knowledge and awareness of DCD is the biggest challenge for their children with DCD in school [44], with one in four parents reporting that their child did not enjoy going to school. Given that adolescents with DCD typically perform worse in school [5], which is associated with higher rates of unemployment and lower wages [3,20,21], raising awareness of DCD among

For peer review only - http://bmjopen.bmj.com/site/about/guidelines.xhtml

educational professionals is essential for children with DCD to receive adequate support. Although most participants with pDCD completed USS and passed their exams, greater understanding and support from teachers and school personnel can significantly improve their academic and long-term outcomes. The participants' repeated assessments throughout their childhood, as a part of the original longitudinal follow-up study, may have helped parents better understand their child's motor difficulties, potentially lessening the negative impact of DCD.

Interaction between DCD and sex

Swedish women have considerably higher rates of graduation from USS and completion of post-secondary education than men [22,25]. When we investigated the educational outcomes within the sexes, we found that less women with pDCD had graduated from USS at 19 years of age compared to women without DCD. In contrast, female NIC recipients without DCD performed similar to, or better than, the general population [22]. A similar pattern was found in the level of education at 28 years of age. Female NIC recipients without DCD had to a larger extent attained a bachelor's or master's degree compared to all other groups.

Our data suggest that DCD may impact men and women differently. While sex and gender differences in neurodevelopmental disorders are increasingly recognized [45], this has not been extensively studied in relation to DCD. A recent study by Cleaton et al. found that women with DCD experienced more gross-motor and non-motor difficulties as adults, and were more affected in their daily activities and participation, than men [46]. Additionally, while children with DCD commonly perform worse academically than their typically developing peers [5,8,10], de Waal et al. found that boys and girls with DCD had different difficulties at school [10]. Factors related to sex and gender, as well as cultural beliefs, may further moderate and mediate the impact of neurodevelopmental disorders and DCD [45,47]. Also, girls with DCD appear to have lower self-competence appraisals compared to boys with DCD in athletic ability, physical appearance, and academic competence [18], and these differences become more pronounced with age. Although our findings are tentative, they contribute to the emerging understanding that DCD affects the sexes differently, which has implications for assessment and management of children with DCD, including possible support in school. Further research is needed to better understand these associations.

Interaction between DCD and attention deficits

Co-occurring neurodevelopmental disorders such as DCD and ADHD can negatively impact various domains, including daily functioning and academic performance [6,7,16]. For

For peer review only

example, a Swedish study reported that 36% of those diagnosed with ADHD and DCD at 6.6 years of age had not graduated from USS in their early thirties [7]. In our study, we observed that 22.7% of the participants with pDCD and an attention deficit at 6.5 years of age had not graduated from USS at age 29 compared to 9.1% of those with DCD alone. Interestingly, we found no significant differences in educational outcomes between those with pDCD with or without a co-occurring attention deficit at 6.5 years of age. This may be because all children met the diagnostic criteria for DCD during childhood, indicating that their daily life was affected by their motor difficulties, and DCD in itself is known to affect educational achievements [5,8,10]. It is also worth noting that the study evaluated attention using CAMPB [27,32], which only measures attention during the assessment and does not consider difficulties in other settings, and the children were not given an ADHD-diagnosis. Additionally, the children's attention was dichotomized into having or not having an attention deficit without considering the type and severity of their attention deficit.

Another possible explanation for not finding differences between the groups is the symptomatic overlap between DCD and ADHD [48,49], which can make it difficult to differentiate between the two disorders at a young age. Moreover, motor development is influenced by the individual, task, and environment [50,51], meaning that each individual's development is unique to their own abilities. Hence, supportive environments that provide opportunities for practice at an individual's level may have a positive effect on outcomes [50,52]. That the children in the original follow-up study participated in repeated assessments may have impacted the parents' knowledge and awareness about their child's difficulties. Additionally, they were guided in how to support and promote motor development and participation, which both may have positively affected outcomes. Furthermore, individuals with DCD and ADHD learn to live with their disorders and develop strategies to overcome their difficulties [41,53]. Lastly, prematurity and low birth weight have been associated with neurodevelopmental disorders, including DCD and ADHD, as well as difficulties with executive functioning and inattention [15,54]. All participants in the study are NIC recipients, and two-thirds were born premature (GW < 37). Prematurity is associated with poorer cognitive and academic outcomes [14,15,55,56], and an increased risk of not reaching curriculum goals during primary school [11,56,57].

Research suggests that children with neurodevelopmental disorders, regardless of whether they were born prematurely or not, are at a higher risk of experiencing difficulties in various domains that are crucial for learning and academic performance. Therefore, any signs of any neurodevelopmental disorder should prompt a thorough evaluation of the child's abilities in all

For peer review only

areas essential for their development and learning [45]. This will enable tailored interventions to optimize development, divert negative pathways, and mitigate negative outcomes.

Strengths and limitations

While this study has several strengths such as its longitudinal design and use of national registers with high coverage and a low rate of missing data, there are also limitations that should be acknowledged. One potential issue is the small sample size, which may have limited the study's ability to detect true differences, especially in subgroup analyses with small group sizes. Therefore, caution should be taken when interpreting the results. Additionally, the study would have benefitted from a population-matched control group and measures of the participants' functioning in adulthood to validate childhood assessments. Furthermore, we only measured educational outcomes as a binary outcome and did not consider grades or support received to graduate, making it difficult to assess the impact of motor difficulties on school performance. Future research should investigate negative events such as lower grades or the resources required to graduate from USS. Regarding educational outcomes, we also lacked access to covariates that are known to affect education, such as socioeconomic background, parental education [17], and birth weight [58], that could have informed our results. Additionally, neurodevelopmental disorders were not specified in the National Patient Registry data, so we could not control for specific disorders. Moreover, the generalizability of our results may be limited by the characteristics of our study sample, as individuals that were healthy in their neonatal period may have had different experiences and outcomes related to DCD. Further, given the huge advances in neonatal care since the late 80-ties, we do not know if this affects outcomes. However, evidence suggests that increased survival rates among the extremely premature has not been accompanied by decreased rates of neurodevelopmental disorders and educational difficulties [15,59,60], making the results relevant today. Finally, it is important to note that the age at follow-up may have affected the results, particularly in terms of the highest level of education, as study participants may continue to graduate later in life.

Conclusions

This study suggests that pDCD during childhood may have lasting impact on educational outcomes into adulthood, particularly among women with pDCD. While further research is needed to determine if these findings apply to the wider DCD-population, they suggest that timely intervention and support for children with motor difficulties could be vital to optimize

For peer review only

learning and development. The findings underscore the importance of early identification and tailored interventions for children with motor difficulties, enabling their academic success throughout their educational journeys. Raising awareness of DCD among parents, healthcare and educational professionals is vital to facilitate early identification and ensure appropriate support and interventions in schools, mitigating the potential negative consequences associated with DCD and promoting positive educational outcomes for children with DCD.

Acknowledgements

We want to acknowledge the children and parents who participated in the initial follow-up study. Additionally, we want to thank Elvira Hemgren (PT), Kristina Persson (PT, PhD) and Bo Strömberg (MD, PhD) for their important work in collecting the baseline data, as well as Jill Zwicker (OT, PhD) and Amanda Kirby (MD, PhD) for their valuable advice during the initial phases of the project.

Author contributions

KJ acquired funding, conceptualized and designed the study with support from FS and RS. IP analysed and managed the data and drafted the initial manuscript. FS, TS, RS and KJ supported data analysis. KJ revised the manuscript. All authors critically reviewed the manuscript, contributed to drafts and approved the final version. We attest all authors meet ICMJE requirements for authorship. KJ acts as guarantor.

Funding

This work was supported by the Gillbergska Foundation (grant/award no. n/a), Uppsala University Hospital through the Medical Training and Research Agreement Funds (ALF) (grant/award no. n/a); and Uppsala University through the Hedström Foundation and the Larsson Foundation (grant/award no. n/a).

Competing interests

None declared.

Patient consent for publication

Not applicable.

Data availability statement

Data cannot be shared publicly because it includes potentially identifying and sensitive patient information, therefore access is limited to by request by the Swedish Ethical Review Authority (reg. no. 2019-04119). Data are available from Uppsala University, Legal Affairs

For peer review only

Division (contact via e-mail: registrator@uu.se), for researchers who meet the criteria for access to confidential data.

Ethics approval statement

Approval was given by the Swedish Ethical Review Authority (reg. no. 2019-04119). The register data were delivered anonymized but linked to the participants via unique identification numbers administered by the Swedish Board of Health and Welfare. All data were stored at the Department of Women's and Children's Health at Uppsala University and on password protected servers held by the University. Data were handled in accordance to the General Data Protection Regulation and Swedish legislation. The processing of personal data was registered in Uppsala University's register of personal data processing and monitored by the Data Protection Officer.

For peer review only

References

1. Blank R, Barnett AL, John Cairney, et al. International clinical practice recommendations on the definition, diagnosis, assessment, intervention, and psychosocial aspects of developmental coordination disorder. Dev Med Child Neurol 2019;61:242-85. https://doi.org/10.1111/dmcn.14132
2. Cleaton MAM, Kirby A. Why do we find it so hard to calculate the burden of neurodevelopmental disorders? J Child Dev Disord 2018;4:10. Doi: 10.4172/2472-1786.100073
3. OECD. Education at a Glance 2021: OECD Indicators. OECD Publishing, Paris. https://doi.org/10.1787/b35a14e5-en
4. Cantell MH, Smyth MM, Ahonen TP. Two distinct pathways for developmental coordination disorder: Persistence and resolution. Hum Mov Sci 2003;22:413–31. https://doi.org/10.1016/j.humov.2003.09.002.
5. Harrowell I, Hollén L, Lingam R, et al. The impact of developmental coordination disorder on educational achievement in secondary school. Res Dev Disabil 2018;72:13–22. https://doi.org/10.1016/j.ridd.2017.10.014
6. Landgren V, Fernell E, Gillberg C, et al. Deficits in attention, motor control and perception childhood to age 30 years: prospective case-control study of outcome predictors. BMJ Open 2022;12:e54424. https://doi.org/10.1136/bmjopen-2021-054424
7. Landgren V, Fernell E, Gillberg C, et al. Attention-deficit/hyperactivity disorder with developmental coordination disorder: 24-year follow-up of a population-based sample. BMC Psychiatry 2021;21:161. https://doi.org/10.1186/s12888-021-03154-w.
8. Lingam R, Golding J, Jongmans MJ, et al. The association between developmental coordination disorder and other developmental traits. Pediatrics 2010;126(5), e1109–e1118. https://doi.org/10.1542/peds.2009-2789
9. Gomez A, Piazza M, Jobert A, et al. Mathematical difficulties in developmental coordination disorder: Symbolic and nonsymbolic number processing. Res Dev Disabil 2015;43–44:167–78. https://doi.org/10.1016/j.ridd.2015.06.011
10. De Waal E, Pienaar AE, Coetzee D. Gender differences in academic achievement of children with developmental coordination disorder. SAJCE 2018;8, a515. https://doi.org/10.4102/sajce.v8i1.515
11. Twilhaar ES, de Kieviet JF, Aarnoudse-Moens CSH, et al. Academic performance of children born preterm: A meta-analysis and meta-regression. Arch Dis Child Fetal Neonatal Ed 2018;103:F322-F330. https://doi.org/10.1136/archdischild-2017-312916
12. Jangmo A, Stålhandske A, Chang Z, et al. Attention-deficit/hyperactivity disorder, school performance, and effect of medication. J Am Acad Child Adolesc Psychiatry 2019;58(4):423-32. https://doi.org/10.1016/j.jaac.2018.11.014
13. Arnold LE, Hodgkins P, Kahle J, et al. Long-Term Outcomes of ADHD: Academic Achievement and Performance. J Atten Disord 2020;24:73–85. https://doi.org/10.1177/1087054714566076
14. Allotey J, Zamora J, Cheong-See F, et al. Cognitive, motor, behavioural and academic performances of children born preterm: a meta-analysis and systematic review involving 64 061 children. BJOG. 2018;125:16–25. https://doi.org/10.1111/1471-0528.14832

For peer review only

15. Doyle LW, Spittle A, Anderson PJ, et al. School-aged neurodevelopmental outcomes for children born extremely preterm. Arch Dis Child. 2021;106:834–38. https://doi.org/10.1136/archdischild-2021-321668.
16. Rasmussen P, Gillberg C. Natural outcome of ADHD with developmental coordination disorder at age 22 years: A controlled, longitudinal, community-based study. J Am Acad Child Adolesc Psychiatry 2000;39:1424–31. https://doi.org/10.1097/00004583-200011000-00017
17. The Swedish National Agency for Education. Analyser av familjebakgrundens betydelse för skolresultaten och skillnader mellan skolor. 2018. Retrieved from: https://www.skolverket.se/publikationsserier/rapporter/2018/analyser-av-familjebakgrundens-betydelse-for-skolresultaten-och-skillnader-mellan-skolor?sv.target=12.3253018117f029df83d13e&sv.12.3253018117f029df83d13e.route=/settings. Accessed June 13, 2022.
18. Brown DMY, Cairney J. The synergistic effect of poor motor coordination, gender and age on self-concept in children: A longitudinal analysis. Res Dev Disabil 2020;98:103576. https://doi.org/10.1016/j.ridd.2020.103576
19. The Swedish National Agency for Education. Overview of the Swedish upper secondary school. 2012. Retrieved from: https://www.skolverket.se/download/18.6bfaca41169863e6a659a16/1553964183391/pdf2748.pdf. Accessed June 13, 2022.
20. Swedish Public Employment Service. 2016. Perspektiv på ungdomsarbetslösheten. Retrieved from: https://arbetsformedlingen.se/download/18.3e623d4f16735f3976ea8e/Perspektiv%20p%C3%A5%20ungdomsarbetsl%C3%B6sheten.pdf. Accessed June 13, 2022.
21. Statistics Sweden. Labour Force Surveys, Grundtabeller årsmedeltal 2020. Retrieved from: https://www.scb.se/hitta-statistik/statistik-efter-amne/arbetsmarknad/arbetskraftsundersokningar/arbetskraftsundersokningarna-aku/pong/tabell-och-diagram/icke-sasongrensade-data/grundtabeller-aku-1574-ar-ar/2020. Accessed June 13, 2022.
22. The Delegation for the Employment of Young People and Newly Arrived Migrants. 2016. Fullföljd gymnasieutbildning. Retrieved from: https://www.dua.se/Fullfoljd-Gymnasieutbildning. Accessed June 13, 2022.
23. Hultkrantz L, Karpaty P, Vimefall E. Education-earnings linkage for assessing societal benefits of interventions for children and youth in Sweden. Psychosocial Intervention 2017;26:171–9. https://doi.org/10.1016/j.psi.2017.06.001
24. Statistics Sweden. Population 16-74 years of age by region, highest level of education, age and sex. Year 1985 - 2020. Retrieved from: https://www.statistikdatabasen.scb.se/pxweb/sv/ssd/START__UF__UF0506/Utbildning/?loadedQueryId=57012&timeType=from&timeValue=2000. Accessed June 13, 2022 2021.
25. Swedish Higher Education Authority. Higher education in Sweden - 2018 status report. 2018. Retrieved from: https://english.uka.se/about-us/publications/reports--guidelines/reports--guidelines/2018-06-29-higher-education-in-sweden---2018-status-report.html. Accessed June 13, 2022.
26. Persson K, Strömberg B. Structured observation of motor performance (SOMP-I) applied to preterm and full term infants who needed neonatal intensive care. Across-

For peer review only

sectional analysis of progress and quality of motor performance at ages 0-10 months. Early Hum Dev 1995;43(3):205–24. https://doi.org/10.1016/0378-3782(95)01678-3

27. Hemgren E, Persson K. Deficits in motor co-ordination and attention at 3 years of age predict motor deviations in 6.5-year-old children who needed neonatal intensive care. Child Care Health Dev 2009;35:120–9. https://doi.org/10.1111/j.1365-2214.2008.00896.x

28. Montgomery C, Johansen K, Lucas S, et al. The Structured Observation of Motor Performance in Infants can detect cerebral palsy early in neonatal intensive care recipients. Early Hum Dev 2017;113:31–9. https://doi.org/10.1016/j.earlhumdev.2017.07.009

29. Stott DH, Moyes EA, Henderson SE. Test of Motor Impairment, Henderson Revision. Brook Educational Publishing. 1984.

30. Henderson SE, Sugden DA. The Movement Assessment Battery for Children. The Psychological Corporation, London, UK 1992.

31. Holle B, Bönnelycke K, Kemp E, et al. Motorisk-perceptuell utveckling. Psykologiförlaget AB. 1977.

32. Hemgren E, Persson K. A Model for Combined Assessment of Motor Performance and Behaviour in 3-year-old Children. Ups J Med Sci 1999;104:49–85. doi:10.3109/03009739909178955

33. American Psychiatric Association (APA). Diagnostic and Statistical Manual of Mental Disorders Fourth Edition Text Revision (DSM-IV-TR). Washington DC: APA 2000. https://doi.org/10.1176/appi.books.9780890423349

34. von Elm E, Altman DG, Egger M, et al. The strengthening the reporting of observational studies in epidemiology (STROBE) statement: guidelines for reporting observational studies. Int J Surg 2014;12:1495–9. doi: 10.1016/j.ijsu.2014.07.013.

35. Statistics Sweden. Longitudinal integrated database for health insurance and labour market studies (LISA). 2022. Retrieved from: https://www.scb.se/en/services/ordering-data-and-statistics/ordering-microdata/vilka-mikrodata-finns/longitudinella-register/longitudinal-integrated-database-for-health-insurance-and-labour-market-studies-lisa/. Accessed June 13, 2022.

36. National Board of Health and Welfare. National Patient Register. 2022. Retrieved from: https://www.socialstyrelsen.se/en/statistics-and-data/registers/national-patient-register/. Accessed June 13, 2022.

37. Brooke HL, Talbäck M, Hörnblad J, et al. The Swedish cause of death register. Eur J Epidemiol 2017;**32**:765–73. https://doi.org/10.1007/s10654-017-0316-1

38. Ludvigsson JF, Svedberg P, Olén O, et al. The longitudinal integrated database for health insurance and labour market studies (LISA) and its use in medical research. Eur J Epidemiol 2019;34:423-37. https://doi.org/10.1007/s10654-019-00511-8

39. Kumar LM, Stephen J, George R, et al. Use of Effect Size in medical research: A brief primer on its why and how. KJP 2022;35:78-82. doi:10.30834/kjp.35.1.2022.322

40. Yngve M, Lidström H, Ekbladh E, et al. Which students need accommodations the most, and to what extent are their needs met by regular upper secondary school? A cross-sectional study among students with special educational needs. Eur J Spec Needs Educ 2019;34:327–41. https://doi.org/10.1080/08856257.2018.1501966

41. Zahlander J. 'I kind of found my own way of doing it': Interviewing adults with motor difficulties as children, a qualitative study. Available from:

For peer review only

http://urn.kb.se/resolve?urn=urn:nbn:se:uu:diva-428885 Accessed May 10, 2023 2020.
42. Hunt J, Zwicker JG, Godecke E, et al. Awareness and knowledge of developmental coordination disorder: A survey of caregivers, teachers, allied health professionals and medical professionals in Australia. Child Care Health Dev 2021;47:174–83. https://doi.org/10.1111/cch.12824
43. Licari MK, Alvares GA, Bernie C, et al. The unmet clinical needs of children with developmental coordination disorder. Pediatr Res 2021;90:826–31. https://doi.org/10.1038/s41390-021-01373-1
44. Licari M, Williams J, The Impact for DCD Team. National Survey Evaluating the Impact of Developmental Coordination Disorder in Australia: Summary of Results. Telethon Kids Institute, Perth, Western Australia 2020. Available from: https://www.telethonkids.org.au/projects/impact-for-dcd/
45. Bölte S, Neufeld J, Marschik PB, et al. Sex and gender in neurodevelopmental conditions. Nat Rev Neurol 2023;19:136–59. https://doi.org/10.1038%2Fs41582-023-00774-6
46. Cleaton MAM, Tal-Saban M, Hill EL, et al. Gender and age differences in the presentation of at-risk or probable Developmental Coordination Disorder in adults. Res Dev Disabil 2021;115:104010. https://doi.org/10.1016/j.ridd.2021.104010
47. Cairney J, Hay J, Faught B, et al. Developmental Coordination Disorder, Self-efficacy toward physical activity, and play: does gender matter? APAQ 2005;22:67-82. https://doi.org/10.1123/apaq.22.1.67
48. Goulardins JB, Rigoli D, Licari M, et al. Attention deficit hyperactivity disorder and developmental coordination disorder: two separate disorders or do they share a common etiology. Behav Brain Res 2015;292:484–92. https://doi.org/10.1016/j.bbr.2015.07.009
49. Meachon EJ, Meyer M, Wilmut K, et al. Evoked potentials differentiate Developmental Coordination Disorder from Attention-Deficit/Hyperactivity Disorder in a stop-signal task: a pilot study. Front Hum Neurosci 2021;15. https://doi.org/10.3389/fnhum.2021.629479
50. Wilmut K. Performance under varying constraints in Developmental Coordination Disorder (DCD): difficulties and compensations. Curr Dev Disord Rep 2017;4:46–52. https://doi.org/10.1007/s40474-017-0108-z
51. Adolph KE, Hoch JE. Motor Development: Embodied, Embedded, Enculturated, and Enabling. Annu Rev Psychol 2019;70:141–64. https://doi.org/10.1146%2Fannurev-psych-010418-102836
52. Smits-Engelsman B, Vinçon S, Blank R, et al. Evaluating the evidence for motor-based interventions in developmental coordination disorder: A systematic review and meta-analysis. Res Dev Disabil 2018;74:72–102. https://doi.org/10.1016/j.ridd.2018.01.002
53. Bjerrum MB, Pedersen PU, Larsen P. Living with symptoms of attention deficit hyperactivity disorder in adulthood: a systematic review of qualitative evidence. JBI Database System Rev Implement Rep 2017;15:1080–153. Doi: 10.11124/JBISRIR-2017-003357
54. Franz AP, Bolat UG, Bolat H, et al. Attention-deficit/hyperactivity disorder and very preterm/very low birth weight: a meta-analysis. 2018;141(1):e20171645. doi: 10.1542/peds.2017-1645

For peer review only

55. Raju TNK, Buist AS, Blaisdell CJ, et al. Adults born preterm: a review of general health and system-specific outcomes. Acta Paediatr 2017;106:1409–37. https://doi.org/10.1111/apa.13880
56. O'Nions E, Wolke D, Johnson S, et al. Preterm birth: Educational and mental health outcomes. Clin Child Psychol Psychiatry. 2021;26:750–9. https://doi.org/10.1177/13591045211006754
57. Twilhaar ES, de Kieviet JF, van Elburg RM, et al. Academic trajectories of very preterm born children at school age. Arch Dis Child Fetal Neonatal Ed 2019;104:F419–23. https://doi.org/10.1136/archdischild-2018-315028
58. Hutchinson EA, de Luca CR, Doyle LW, et al. School-age outcomes of extremely preterm or extremely low birth weight children. Pediatrics 2013;131(4):e1053–e1061. https://doi.org/10.1542/peds.2012-2311
59. Marlow N, Ni Y, Lancaster R, et al. No change in neurodevelopment at 11 years after extremely preterm birth. Arch Dis Child Fetal Neonatal Ed 2021;106(4):418-24. https://doi.org/10.1136/archdischild-2020-320650
60. Morsing E, Lundgren P, Hård AL, et al. Neurodevelopmental disorders and somatic diagnoses in a national cohort of children born before 24 weeks of gestation. Acta Paediatr 2022;111(6):1167–75. https://doi.org/10.1111/apa.16316

For peer review only

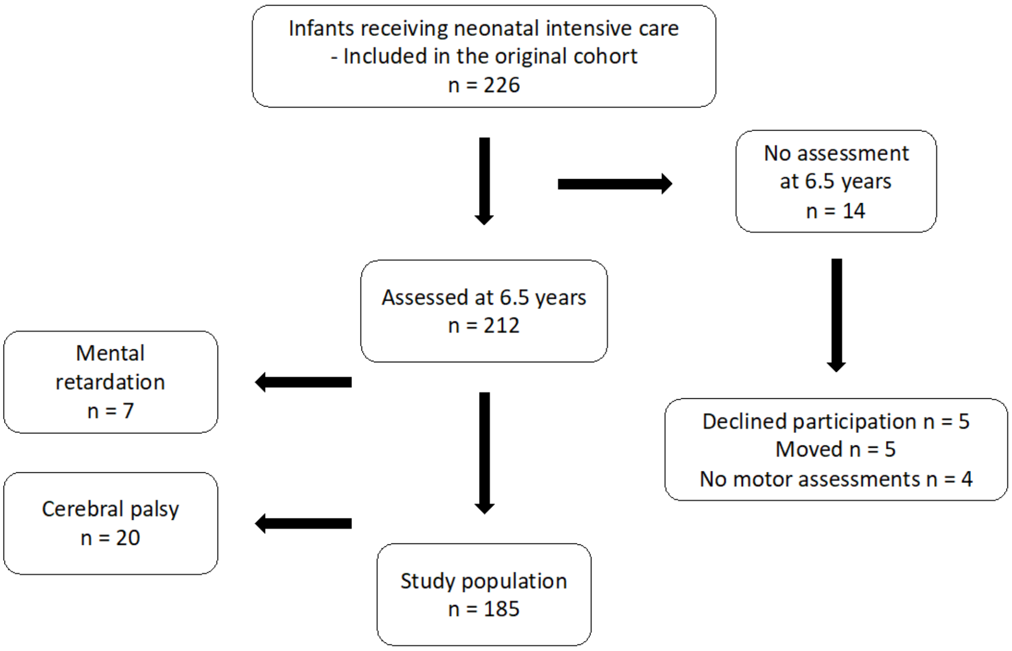

Supplementary file 1. Flow chart of recruitment.

Supplementary file 2. ICD-8/9 codes included in the variable "Diagnoses posing a risk of motor problems".

ICD-code	Diagnosis
040–046	Poliomyelitis and other enterovirus diseases of central nervous system (ICD-8)
190–199	Malignant neoplasm of other and unspecified sites (ICD-8 and ICD-9)
210–228	Benign neoplasms (ICD-8)
300–316	Neurotic disorders, personality disorders, and other nonpsychotic mental disorders (ICD-8)
320–324	Inflammatory diseases of central nervous system (ICD-8)
330–337	Hereditary and degenerative diseases of the central nervous system (ICD-9)
340–349	Other diseases of central nervous system (ICD-8)
430–438	Cerebrovascular disease (ICD-8 and ICD-9)
710–719	Arthropathies and related disorders (ICD-9)
740–759	Congenital anomalies (ICD-8 and ICD-9)
760–779	Certain conditions originating in the perinatal period (ICD-9) or certain causes of perinatal morbidity and mortality (ICD-8)
780–789	Symptoms referable to systems or organs (ICD-8)
850–854	Intracranial injury, excluding those with skull fracture (ICD-9)

Note: ICD = International Statistical Classification of Diseases and Related Health Problems - Eight/Ninth revision

For peer review only

Supplementary file 3. Number of neurodevelopmental disorders registered in specialized outpatient care.

**ICD-10 Codes**		n (%)
F80-F89	Disorders of psychological development	2 (1.1)
F90-F98	Behavioral and emotional disorders with onset usually occurring in childhood and adolescence	10 (5.5)
R47-R49	Symptoms and signs involving speech and voice	2 (1.1)

F80-F89 includes: Developmental Coordination Disorder, Developmental language disorder, Dyslexia, Autism Spectrum Disorders and impairments in reading, writing and arithmetic.

F90-F98 includes: Attention Deficit Hyperactivity Disorder and Tic disorders.

R47-R49 includes: Dyslexia.

Note: ICD=International Statistical Classification of Diseases and Related Health Problems.

For peer review only

Supplementary file 4. Educational outcomes for men and women with or without pDCD.

	Total n=183				No DCD n=138				DCD n=45			
	**Men** n (%) 108 (59.0)	**Women** n (%) 75 (41.0)	**P**	***g***	**Men** n (%) 80 (58.0)	**Women** n (%) 58 (48.0)	**P**	***g***	**Men** n (%) 28 (62.2)	**Women** n (%) 17 (37.8)	**P**	***g***
**Age (yrs) at graduation from USS**												
Median	19.0	19.0	.376		19.0	19.0	.342		19.0	19.0	.882	
Min-max	19.0–26.0	19.0–26.0			19.0–22.0	19.0–26.0			19.0–26.0	19.0–24.0		
IQR	19.0-19.0	19.0-19.0			19.0-19.0	19.0-19.0			19.0-20.0	19.0-20.0		
Mean (SD)	19.3 (.9)	19.3 (1.1)		0.00	19.2 (.6)	19.2 (1.0)		0.00	19.6 (1.4)	19.8 (1.5)		0.14
Missing data, total	10 (9.3)	8 (10.7)			6 (7.5)	3 (5.2)			4 (14.3)	5 (29.4)		
No graduation	10 (9.3)	6 (8.0)	.788		6 (7.5)	3 (5.2)	.734		4 (14.3)	3 (17.7)	.692	
Absent/incomplete register data		2 (2.7)								2 (11.8)		
**USS graduation at 19 yrs**												
Yes	76 (70.4)	56 (75.7)	.431		60 (75.0)	48 (82.8)	.275		16 (57.1)	8 (50.0)	.647	
No	32 (29.6)	18 (24.3)			20 (25.0)	10 (17.2)			12 (42.9)	8 (50.0)		
Missing data		1 (1.3)								1 (5.9)		
**USS graduation at 24 yrs**												
Yes	97 (89.8)	67 (90.5)	.872		74 (92.5)	54 (93.1)	1.000		23 (82.1)	13 (81.3)	.941	
No	11 (10.2)	7 (9.5)			6 (7.5)	4 (6.9)			5 (17.9)	3 (18.8)		
Missing data		1 (1.3)								1 (5.9)		
**USS graduation at 29 yrs**												
Yes	98 (90.7)	68 (91.9)	.788		74 (92.5)	55 (94.8)	.734		24 (85.7)	13 (81.3)	.697	
No	10 (9.3)	6 (8.1)			6 (7.5)	3 (5.2)			4 (14.3)	3 (18.8)		
Missing data		1 (1.3)								1 (5.9)		
**Level of education at 28 yrs**												
Compulsory school, 9 yrs	6 (5.7)	4 (5.6)	.073		4 (5.1)	3 (5.2)	.058		2 (7.4)	1 (7.1)	.880	
Upper secondary education, ≤2 yrs	4 (3.8)	2 (2.8)			2 (2.5)	0 (0)			2 (7.4)	2 (14.3)		
Upper secondary education, 3 yrs	52 (49.1)	22 (30.6)			38 (48.1)	17 (29.3)			14 (51.9)	5 (35.7)		
Post-secondary education, <3 yrs	17 (16.0)	12 (16.7)			14 (17.7)	10 (17.2)			3 (11.1)	2 (14.3)		
Bachelor's or master's degree	27 (25.5)	32 (44.4)			21 (26.6)	28 (42.8)			6 (22.2)	4 (28.6)		
Missing data	1 (.9)	4 (5.3)			1 (1.3)				1 (3.6)	3 (17.6)		

Note: DCD=Developmental Coordination Disorder; IQR=interquartile range; Min=minimum; Max=maximum; USS=Upper Secondary School, Yrs=Years.

For peer review only

Reporting checklist for cohort study.

Based on the STROBE cohort guidelines.

Instructions to authors

Complete this checklist by entering the page numbers from your manuscript where readers will find each of the items listed below.

Your article may not currently address all the items on the checklist. Please modify your text to include the missing information. If you are certain that an item does not apply, please write "n/a" and provide a short explanation.

Upload your completed checklist as an extra file when you submit to a journal.

In your methods section, say that you used the STROBE cohortreporting guidelines, and cite them as:

von Elm E, Altman DG, Egger M, Pocock SJ, Gotzsche PC, Vandenbroucke JP. The Strengthening the Reporting of Observational Studies in Epidemiology (STROBE) Statement: guidelines for reporting observational studies.

		Reporting Item	Page Number
**Title and abstract**			
Title	#1a	Indicate the study’s design with a commonly used term in the title or the abstract	1

For peer review only

Abstract	#1b	Provide in the abstract an informative and balanced summary of what was done and what was found	2
**Introduction**			
Background / rationale	#2	Explain the scientific background and rationale for the investigation being reported	4-5
Objectives	#3	State specific objectives, including any prespecified hypotheses	5
**Methods**			
Study design	#4	Present key elements of study design early in the paper	5-6
Setting	#5	Describe the setting, locations, and relevant dates, including periods of recruitment, exposure, follow-up, and data collection	5-6
Eligibility criteria	#6a	Give the eligibility criteria, and the sources and methods of selection of participants. Describe methods of follow-up.	5-6
Eligibility criteria	#6b	For matched studies, give matching criteria and number of exposed and unexposed	n/a
Variables	#7	Clearly define all outcomes, exposures, predictors, potential confounders, and effect modifiers. Give diagnostic criteria, if applicable	6-7
Data sources / measurement	#8	For each variable of interest give sources of data and details of methods of assessment (measurement). Describe comparability of assessment methods if there is more than	6

For peer review only

		one group. Give information separately for for exposed and unexposed groups if applicable.	
Bias	#9	Describe any efforts to address potential sources of bias	7
Study size	#10	Explain how the study size was arrived at	5
Quantitative variables	#11	Explain how quantitative variables were handled in the analyses. If applicable, describe which groupings were chosen, and why	6-8
Statistical methods	#12a	Describe all statistical methods, including those used to control for confounding	8
Statistical methods	#12b	Describe any methods used to examine subgroups and interactions	8
Statistical methods	#12c	Explain how missing data were addressed	7
Statistical methods	#12d	If applicable, explain how loss to follow-up was addressed	n/a
Statistical methods	#12e	Describe any sensitivity analyses	n/a

For peer review only

Results

Participants	#13a	Report numbers of individuals at each stage of study—eg numbers potentially eligible, examined for eligibility, confirmed eligible, included in the study, completing follow-up, and analysed. Give information separately for for exposed and unexposed groups if applicable.	5
Participants	#13b	Give reasons for non-participation at each stage	5-6
Participants	#13c	Consider use of a flow diagram	Supplementary file 1
Descriptive data	#14a	Give characteristics of study participants (eg demographic, clinical, social) and information on exposures and potential confounders. Give information separately for exposed and unexposed groups if applicable.	8-9
Descriptive data	#14b	Indicate number of participants with missing data for each variable of interest	Page 7, and table 2, 4 and 5
Descriptive data	#14c	Summarise follow-up time (eg, average and total amount)	5-6
Outcome data	#15	Report numbers of outcome events or summary measures over time. Give information separately for exposed and unexposed groups if applicable.	10 and table 2-3
Main results	#16a	Give unadjusted estimates and, if applicable, confounder-adjusted estimates and their precision (eg,	Table 3

For peer review only

		95% confidence interval). Make clear which confounders were adjusted for and why they were included	
Main results	#16b	Report category boundaries when continuous variables were categorized	Table 1-5
Main results	#16c	If relevant, consider translating estimates of relative risk into absolute risk for a meaningful time period	n/a
Other analyses	#17	Report other analyses done—eg analyses of subgroups and interactions, and sensitivity analyses	12-14 and table 4-5
**Discussion**			
Key results	#18	Summarise key results with reference to study objectives	14-15
Limitations	#19	Discuss limitations of the study, taking into account sources of potential bias or imprecision. Discuss both direction and magnitude of any potential bias.	18
Interpretation	#20	Give a cautious overall interpretation considering objectives, limitations, multiplicity of analyses, results from similar studies, and other relevant evidence.	14-18
Generalisability	#21	Discuss the generalisability (external validity) of the study results	15-18
**Other Information**			

For peer review only

Funding	#22	Give the source of funding and the role of the funders for the present study and, if applicable, for the original study on which the present article is based	19

Notes:

- 14b: 7, and table 2, 4 and 5 The STROBE checklist is distributed under the terms of the Creative Commons Attribution License CC-BY. This checklist was completed on 28. December 2022 using https://www.goodreports.org/, a tool made by the EQUATOR Network in collaboration with Penelope.ai

For peer review only
